# Cooperation between Epstein-Barr Virus Immune Evasion Proteins Spreads Protection from CD8^+^ T Cell Recognition across All Three Phases of the Lytic Cycle

**DOI:** 10.1371/journal.ppat.1004322

**Published:** 2014-08-21

**Authors:** Laura L. Quinn, Jianmin Zuo, Rachel J. M. Abbott, Claire Shannon-Lowe, Rosemary J. Tierney, Andrew D. Hislop, Martin Rowe

**Affiliations:** School of Cancer Sciences and Centre for Human Virology, University of Birmingham College of Medical and Dental Sciences, Edgbaston, Birmingham, United Kingdom; Baylor College of Medicine, United States of America

## Abstract

CD8^+^ T cell responses to Epstein-Barr virus (EBV) lytic cycle expressed antigens display a hierarchy of immunodominance, in which responses to epitopes of immediate-early (IE) and some early (E) antigens are more frequently observed than responses to epitopes of late (L) expressed antigens. It has been proposed that this hierarchy, which correlates with the phase-specific efficiency of antigen presentation, may be due to the influence of viral immune-evasion genes. At least three EBV-encoded genes, BNLF2a, BGLF5 and BILF1, have the potential to inhibit processing and presentation of CD8^+^ T cell epitopes. Here we examined the relative contribution of these genes to modulation of CD8^+^ T cell recognition of EBV lytic antigens expressed at different phases of the replication cycle in EBV-transformed B-cells (LCLs) which spontaneously reactivate lytic cycle. Selective shRNA-mediated knockdown of BNLF2a expression led to more efficient recognition of immediate-early (IE)- and early (E)-derived epitopes by CD8^+^ T cells, while knock down of BILF1 increased recognition of epitopes from E and late (L)-expressed antigens. Contrary to what might have been predicted from previous ectopic expression studies in EBV-negative model cell lines, the shRNA-mediated inhibition of BGLF5 expression in LCLs showed only modest, if any, increase in recognition of epitopes expressed in any phase of lytic cycle. These data indicate that whilst BNLF2a interferes with antigen presentation with diminishing efficiency as lytic cycle progresses (IE>E>>L), interference by BILF1 increases with progression through lytic cycle (IE<E<<L). Moreover, double-knockdown experiments showed that BILF1 and BNLF2a co-operate to further inhibit antigen presentation of L epitopes. Together, these data firstly indicate which potential immune-evasion functions are actually relevant in the context of lytic virus replication, and secondly identify lytic-cycle phase-specific effects that provide mechanistic insight into the immunodominance pattern seen for CD8^+^ T cell responses to EBV lytic antigens.

## Author Summary

Epstein Barr Virus (EBV), an oncogenic herpesvirus, infects and persists asymptomatically in the majority of humans. In immunocompetent individuals, EBV co-exists with its host as a lifelong infection in the face of strong anti-viral CD8^+^ T-cell responses. Evasion of this immune-response is presumed to be due in part to immune-modulating mechanisms of certain EBV-encoded proteins expressed during lytic cycle replication. Three such proteins (BNLF2a, BGLF5 and BILF1) have been identified biochemically as able to interfere with HLA-class I antigen presentation. In this study we investigated these proteins in the context of EBV-infected cells in lytic cycle, and their functional recognition by EBV virus-specific CD8^+^ T-cells. A novel feature of EBV biology was revealed; rather than demonstrating simple redundancy, evasion proteins effect optimum temporal protection at different phases of lytic cycle. BNLF2a strongly inhibited CD8^+^ T-cell recognition immediately after the EBV-infected cells entered lytic cycle, with its influence waning upon progression to later phases of lytic cycle. Conversely, BILF1 strongly inhibited recognition predominantly at the late phase of lytic cycle. Unexpectedly, despite its well-characterised molecular functions, BGLF5 had relatively little effect on recognition at any stage of lytic cycle. Our results help to explain the previously-identified unusual pattern of immunodominance of anti-EBV CD8^+^ T-cell responses.

## Introduction

Members of the human herpes family of viruses have co-evolved with their hosts to persist as largely asymptomatic, latent infections. However, under conditions of immune T cell impairment as seen for example in immunosuppressed transplant recipients, herpesviruses may reactivate, often with clinical symptoms [1]–[4]. This reflects the vital role of T cell-mediated immune responses in controlling, albeit not eliminating, persistent herpesvirus infections [5]–[8]. The ability of these viruses to persist and be transmitted by the immune host is achieved through two strategies: firstly, the establishment of a latent infection with minimal if any viral antigen expression in long lived cell types, and secondly, the synthesis of viral proteins that interfere with antigen processing pathways in the infected cell during the virus-productive phase of replication. Multiple immune evasion proteins have been identified within herpesviruses of the α and β families (e.g., herpes simplex virus, HSV, and cytomegalovirus, CMV, respectively) and these proteins have been shown to cooperate with each other during lytic cycle replication of the individual viruses. Whether the γ-herpesvirus immune evasion mechanisms similarly cooperate with each other is unknown.

The prototypic human γ-herpesvirus, Epstein-Barr virus (EBV), establishes latency in the memory B lymphocyte pool [9]. Studies of infectious mononucleosis patients suggest that during primary infection, EBV seeds this compartment as a reservoir of infected cells by inducing a growth-transforming infection of B lymphocytes through the coordinated expression of 8 transformation-associated proteins [9]. Upon establishment of virus persistence, such growth-transformed cells are well controlled by latent antigen-specific CD8^+^ T cells, and the virus is maintained in a latent and immunologically silent state in resting B cells. Periodically the virus reactivates into its lytic or virus productive phase of replication to allow infection of new cells and transmission to other hosts. Lytic replication is characterized by the sequential expression of two immediate-early (IE) genes (*BZLF1* and *BRLF1*), around 30 early (E) genes followed by around 30 late (L) genes. This provides a potentially diverse repertoire of antigens for immune targeting and strong responses are made to epitopes drawn from the immediate early and some early expressed antigens. A testament to the efficacy of the lytic and latent epitope-specific CD8^+^ T cell responses is that although 90% of adults worldwide are infected with EBV, infection remains largely asymptomatic [10]. However, high levels of viral particles have been proposed to be synthesised and shed in such immune hosts [11]. Additionally *in vitro* models show that in the absence of immune effectors, B cells reactivating from latency in to lytic cycle can remain viable and go on producing virus for several days [12]. *In vivo* therefore, T cell recognition within this extended window of replication has the potential to limit virus production, and evading recognition would clearly be of an advantage to the virus in increasing its chances of transmission from the virus-carrying host.

Following the observation that HLA-class I expression at the cell surface of EBV-infected cells was decreased upon entry into lytic cycle [13], it was also demonstrated that there was increasing evasion of CD8^+^ T cell recognition by cells replicating EBV as they progressed through lytic cycle [14]. Thus EBV-specific CD8^+^ T cells which targeted antigens expressed in the IE wave of expression recognised their target epitopes relatively well, while CD8^+^ T cells specific for E expressed proteins recognised their target epitopes at an intermediate level, and L epitope-specific effectors were relatively poor at recognising their targets.

Subsequently, three EBV lytic cycle genes were shown by ectopic expression in EBV-negative cell models to encode proteins that interfere with the HLA class I antigen processing pathway [15]–[18]. These proteins are: BNLF2a, which associates with the Transporter associated with Antigen Processing (TAP) to block translocation of peptide fragments from the cytosol to the endoplasmic reticulum, thus preventing their access to HLA class I molecules [15], [19]–[21]; BGLF5, which encodes an exonuclease that degrades mRNA and thus reduces global levels of host cell transcripts, including those for HLA and TAP [17], [22], [23]; and BILF1, which binds to HLA class I/peptide complexes and both interferes with their transport to the cell surface and increases the turnover of pre-existing cell-surface HLA class I/peptide complexes, targeting them for lysosomal degradation [16], [24], [25].

Although the individual EBV evasion genes have been well-studied in model systems, little is known about their contributions to evasion in the context of natural EBV lytic cycle. The limited information available suggests that BNLF2a may only be effective during the IE- and E-phases of lytic cycle [19], and yet cells in the L-phase show greatest resistance to EBV-specific CD8^+^ T cells. To better understand why L-phase viral antigens are less immunogenic, we have knocked-down BNLF2a, BGLF5 and BILF1 expression in spontaneously lytic LCLs and examined the efficiency of recognition of these cells by IE, E and L antigen-specific CD8^+^ T cell clones. The data show that of these three gene products, BNLF2a and BILF1 are the major effectors of evasion and they cooperate to provide immune protection across all three phases of the lytic cycle.

## Results

### Generation of BNLF2a, BGLF5 and BILF1 knockdown-LCLs

A panel of EBV transformed B-cell lines (LCLs) from suitable HLA-typed donors was first selected in which more than 1% of cells expressed the lytic switch protein BZLF1 as detected by intracellular staining and flow cytometry. These lines therefore contained significant numbers of cells spontaneously entering into lytic cycle, allowing them to be used as targets in T cell recognition assays.

To examine the relative contribution of BNLF2a, BGLF5 and BILF1 to the inhibition of CD8^+^ T cell recognition of EBV infected B cells during lytic cycle we devised a strategy to knockdown the expression of these genes in LCLs using a lentivirus-delivered shRNA. Sequences for these shRNAs were identified by screening candidate siRNA sequences for their ability to silence ectopic expression of BNLF2a, BGLF5 and BILF1 in model systems (data not shown) and incorporating the selected sequences into shRNA lentiviral expression vectors. Each lentivirus expressed a puromycin resistance gene to enable antibiotic enrichment of transduced cells, and a fluorescent tag to monitor transduction efficiency (Table 1).

**Table 1:** shRNA lentivirus vector constructs used.

Vector	Gene target	DNA sequence 5′-3′
pLKO.1-puro-CMV-TagCFP	BNLF2a	CACAGAGTACCACCAGGAG
pLKO.1-puro-CMV-TagFP635	BGLF5	GTGGATTGATGAAGATGTT
pLKO.1-puro-CMV-TagYFP	BILF1	CGAGAACTCCTGAATCATT
pLKO.1-puro-CMV-TagCFP	None	TCCTAAGGTTAAGTCGCCCTC

**Figure 1:**
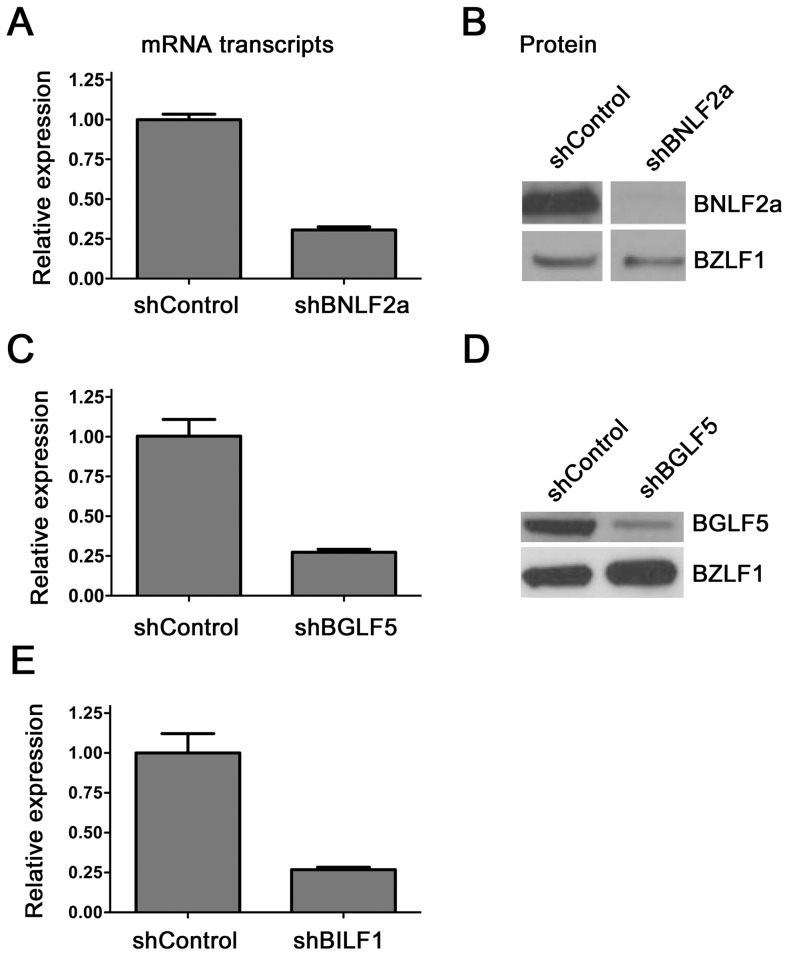
Knockdown of BNL2a, BILF1 and BGLF5 in transduced LCLs. A) qRT-PCR was performed to measure the relative knockdown of BNLF2a transcript levels in shControl- and shBNLF2a-LCLs. BNLF2a-mRNA expression was normalized against BZLF1 and shown as relative BNLF2a expression. (B) BNLF2a protein knockdown was assessed using western blot analysis. Protein levels of BNLF2a and BZLF1 was measured in shControl- and shBNLF2a- LCLs. C) qRT-PCR assay of BGLF5 expression normalized against BZLF1 transcript level. Data are shown as BGLF5 expression relative to shControl LCLs. D) BGLF5 knockdown was confirmed at the protein level using western blot analysis. The expression of BGLF5 and BZLF1 protein was measured in shControl-LCLs and shBGLF5-LCLs. E) qRT-PCR assay of BILF1 expression normalized against BZLF1 transcript. Data are shown as BILF1 expression relative to that in shControl-LCLs.

The efficiency of BNLF2a, BGLF5 and BILF1 knockdown in LCLs using respective shRNA-lentiviruses was first examined by measuring transcript levels by qRT-PCR and protein levels by western blot where relevant antibodies were available. Figure 1a shows a representative example of the relative level of BNLF2a transcript knockdown in shBNLF2a-transduced LCLs compared to non-target shRNA (shControl)-transduced LCLs. As lytic cycle entry is spontaneous and the frequency of entry is unique to individual LCL cultures, differences in the frequency of cells spontaneously undergoing lytic cycle replication in the two LCL lines were accounted for by relating BNLF2a transcript levels to those of the IE lytic BZLF1 transcripts. In all shBNLF2a-transduced LCLs used in this study, the median knockdown of BNLF2a transcripts was 80% (range 70–85%). The knockdown of BNLF2a transcripts (Figure 1A) corresponded to a reduction in BNLF2a protein expression in these same transduced LCLs (Figure 1B). Similar efficiencies of knockdown of BGLF5 transcripts (Figure 1C; median knockdown = 75%, range = 70–85%) and BILF1 transcripts (Figure 1E; median knockdown = 80%, range = 75–90%) were observed in replicate experiments. The lack of available antibodies to detect BILF1 precluded confirmation of knockdown for this EBV protein, but antibodies to BGLF5 confirmed efficient knockdown of BGLF5 protein in LCLs transduced with shBGLF5 (Figure 1D).

### Effect of BNLF2a on CD8^+^ T cell recognition during the IE, E, and L-phases of EBV lytic cycle

To investigate the effect of BNLF2a on epitope presentation during the IE, E and L-phases of lytic cycle, pairs of LCLs transduced with either the shControl- or shBNLF2a-lentiviruses were established from a range of different donors with HLA allele matches for HLA-A2 and/or HLA-B7. These LCLs were used as targets for panels of effector CD8^+^ T cell clones restricted through HLA-A2 or HLA-B7 and specific to epitopes generated during the IE, E and L-phases of lytic cycle, as shown in Table 2.

**Table 2:** Target specificity of T cells used in recognition assays.

Phase of antigen expression	EBV target antigen	Peptide epitope	HLA restriction	No. of clones
Immediate early	BRLF1	YVLDHLIVV	A2	3
Immediate early	BZLF1	DPYQVPFVQAF	B7	1
Early	BMLF1	GLCTLVAML	A2	3
Early	BMRF1	TLDYKPLSV	A2	2
Early	BNLF2b	RPGRPLAGFYA	B7	1
Late	BALF4	FLDKGTYTL	A2	2
Late	BNRF1	WQWEHIPPA	A2	2
Late	BNRF1	YPRNPTWQGNI	B7	1

**Figure 2:**
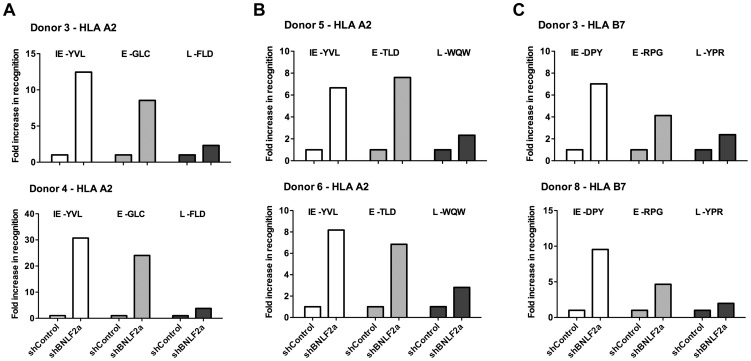
LCLs lacking in BNLF2a expression show increased presentation of epitopes derived from immediate early and early lytic antigens. A) Donor 3 and 4 shBNLF2a-LCLs were used as targets for HLA-A2 restricted effector T cells specific to the YVL epitope of the IE gene BRLF1, the GLC epitope derived from an E gene BMLF1 and the FLD epitope which originates from the L expressed gene BALF4. Recognition was measured by ELISA for IFN- γ released by effector T cells. B) Donor 5 and 6 shBNLF2a-LCLs were used as targets for HLA-A2 restricted effector T cells specific to the YVL epitope of the IE gene BRLF1, the TLD epitope derived from an E gene BRLF1 and the WQW epitope which originates from the L expressed gene BNRF1. C) Donor 3 and 8 shBNLF2a-LCLs were used as targets for HLA-B7 restricted effector T cells specific to the DPY epitope of the IE gene BZLF1, the RPG epitope derived from an E gene BNLF2b and the YPR epitope which originates from the L expressed gene BNRF1. All representative data are shown as fold increase in recognition of shBNLF2a-LCLs compared to shControl transduced LCL counterparts, following normalisation of T cell recognition (IFN-γ release) against the expression levels of the antigen from which each epitope is derived.

T cell recognition was assayed by co-incubation of LCL targets with effector T cells for 18 hours, then measuring by ELISA the amount of IFN-γ released into the supernatant by the T cells. To account for differing levels of spontaneous lytic cycle in the LCLs pairs, and potential indirect effects of knockdown of one lytic gene on other lytic cycle genes, the measured level of T cell recognition was adjusted according to the amount of each target antigen. Levels of target antigen were assayed by measuring their transcript levels, and not protein levels, as target peptides are predominantly derived from defective ribosomal products (DRiPs) rather than mature proteins. The raw T cell function and antigen expression data corresponding to the normalised results in Figure 2 are provided in Supplementary Information (Figures S2–S5). To facilitate comparison between different target/effector T cell combinations, T cell recognition of shBNLF2a LCLs was expressed relative to the amount of recognition of shControl (non-target, scrambled shRNA) after adjusting for differences in target antigen expression. Thus, in Figure S2A, recognition of the shBNLF2a LCL by BRLF1-specific T cells is about 18-fold better than recognition of the control LCL, but as expression of BRLF1 is about 50% higher in the shBNLF2a LCL (Figure S2C), the normalised increase in T cell recognition is reduced to 12.5-fold (Figure S2C).

Figure 2 shows data from six representative experiments using shBNLF2a-LCLs from 5 donors to examine the effect BNLF2a has on HLA-A2 restricted (Figures 2A, 2B) and HLA-B7 restricted (Figure 2C) epitope presentation during IE (hollow bars), E (gray bars) and L stages (black bars) of lytic cycle. As shown in figure 2A (upper graph), BNLF2a-knockdown in donor 3 LCLs (shBNLF2a-LCLs) resulted in 13-fold better recognition of the YVL epitope originating from the BRLF1 IE antigen compared to shControl-LCLs. There was a lower but still substantial 9-fold increase in recognition of the GLC epitope of the BMLF1 E antigen in shBNLF2a-LCLs, and a marginal 2-fold increase in the recognition of the FLD epitope of the BALF4 L antigen. This panel of effector T cells was assayed on donor 4 target LCLs (Figure 2A, lower graph) with the same pattern of increased recognition of IE>E>>L epitopes being reproduced, albeit with different magnitudes of increased recognition. Another panel of HLA-A2 restricted T cells, specific for IE (YVL epitope of BRLF1), E (TLD epitope of BMRF1) and L (WQW epitope of BNRF1) antigens gave a similar pattern of increased recognition of IE, E and L derived epitopes in shBNLF2a-LCLs relative to shControl-LCLs derived from different donors (Figure 2B).

Recognition experiments were also performed using a panel of HLA-B7 restricted T cells recognising the DPY epitope derived from the BZLF1 IE antigen, the RPG epitope of the BNLF2b E antigen and the YPR epitope of the BNRF1 L antigen. As shown in Figure 2C, the pattern of recognition paralleled what was observed with HLA-A2 restricted epitopes; i.e. the reduction of BNLF2a expression led to a pattern of increased recognition of IE>E>>L antigens.

These experiments were repeated and extended into other donors, a summary of which is provided in Table 3. It should be noted the data in this table include some experiments in which it was not possible to determine recognition of the LCLs by a complete panel of IE, E and L specific T cells in parallel assays and so only one or two such T cell specificities were used. Together, these data are consistent with the interpretation that inhibition of TAP-mediated peptide transport into the ER by BNLF2a is more dominant during the IE and E phases of lytic cycle, and that BNLF2a appears to have a much weaker effect at the L stage of lytic cycle.

**Table 3:** Summary of fold increase in CD8^+^ T cell recognition of EBV-antigens presented by shBNLF2a-LCLs compared to shControl-LCLs.

					Fold increase	
Expression phase of target antigen	Target antigen	Epitope	HLA restriction	Number of experiments*	Range	Median
IE	BRLF1	YVL	A2	11	6.5–30.7	17
IE	BZLF1	DPY	B7	4	7–14	8.3
E	BMLF1	GLC	A2	10	7–24	11.5
E	BMRF1	TLD	A2	5	7.5–12	9
E	BNLF2b	RPG	B7	4	4.1–7	5.5
L	BALF4	FLD	A2	11	2–5	2.3
L	BNRF1	WQW	A2	4	2.3–3	2.5
L	BNRF1	YPR	B7	4	2–3.5	2.5

* more than one effector clone specific for each epitope was used where possible. In total, seven different donor LCLs were used.

### BGLF5 plays a minimal role in interfering with CD8^+^ T cell recognition during lytic cycle

**Figure 3:**
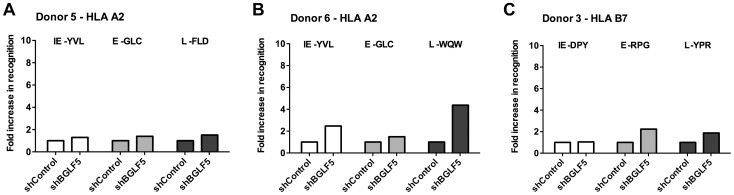
BGLF5 knockdown results in minimal increases in epitope recognition. A) Relative recognition of donor 5 shBGLF5-LCLs, compared to shControl-LCLs, by a panel of HLA-A2 restricted CD8^+^ T cells specific for IE-YVL (BRLF1), E-GLC (BMLF1) and L-FLD (BALF4) epitopes. (B) Relative recognition of donor 6 shBGLF5-LCLs, compared to shControl-LCLs, by a panel of HLA-A2 restricted CD8^+^ T cells specific for IE-YVL (BRLF1), E-GLC (BMLF1) and L-WQW (BNRF1) epitopes. (C) Relative recognition of donor 3 HLA-B7 positive shBGLF5-LCLs, compared to Control LCLs, by HLA-B7 restricted T cells specific for the IE-DPY (BZLF1), E- RPG (BNLF2b) and L-YPR (BNRF1) epitopes. All representative data are shown as fold increase in recognition of shBGLF5-LCLs compared to shControl transduced LCL counterparts, following normalisation of T cell recognition (IFN-γ release) against the expression levels of the antigen from which each epitope is derived.

The role of BGLF5 in interfering with epitope presentation during lytic cycle was then similarly investigated using BGLF5 knockdown LCLs as targets for T cells specific to IE, E and L lytic epitopes. As shown in Figures 3A–C, the knockdown of BGLF5 resulted in a modest, if any, increase in recognition of the IE-YVL and -DPY epitopes that was never more than three times above shControl. Similarly, the increase in recognition of E epitopes in the absence of BGLF5 was not more than 2-fold. Although there is a hint that the knockdown of BGLF5 increased recognition of the L-WQW epitope, this was not reproducible for all L-epitopes and donors in replicate experiments. A summary of all experiments performed is provided in Table 4. Overall, these data suggest that relative to BNLF2a, BGLF5 plays a rather minimal role in interfering with antigen presentation during lytic cycle, contributing only a small effect across all stages of lytic cycle.

**Table 4:** Summary of fold increase in CD8 T cell recognition of EBV-antigens presented by shBGLF5-LCLs compared to shControl-LCLs.

					Fold increase	
Expression phase of target antigen	Target antigen	Epitope	HLA restriction	Number of experiments*	Range	Median
IE	BRLF1	YVL	A2	10	<1–2.5	1.3
IE	BZLF1	DPY	B7	2	1.1–1.4	1.2
E	BMLF1	GLC	A2	8	<1–4	1.6
E	BMRF1	TLD	A2	3	1.4–1.7	1.5
E	BNLF2b	RPG	B7	2	1.9–2.2	2
L	BALF4	FLD	A2	4	1.5–3	1.8
L	BNRF1	WQW	A2	3	1.9–4.3	2.6
L	BNRF1	YPR	B7	3	1.5–1.9	1.9

* more than one effector clone specific for each epitope was used where possible. In total, six different donor LCLs were used.

### BILF1 plays a more dominant role in interfering with CD8^+^ T cell recognition at the late stage of EBV lytic cycle

We next examined the effect that BILF1 expression had on CD8^+^ T cell recognition of IE, E and L lytic epitopes using similar experimental approaches as described above. To this end, shBILF1-LCLs were generated and used as targets for T cells specific for epitopes drawn from the IE, E and L-phases of lytic cycle.

**Figure 4:**
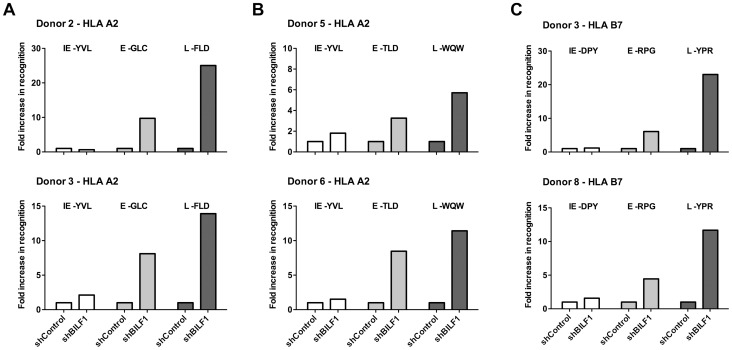
BILF1 predominantly interferes with peptide presentation to CD8^+^ T cells during late stage lytic cycle. A) Donor 2 and 3 shBILF1-LCLs were used as targets for HLA-A2 restricted effector T cells specific to the YVL epitope of the IE gene BRLF1, the GLC epitope derived from an E gene BMLF1 and the FLD epitope which originates from the L expressed gene BALF4. B) Donor 5 and 6 shBILF1-LCLs were used as targets for HLA-A2 restricted effector T cells specific to the YVL epitope of the IE gene BRLF1, the TLD epitope derived from an E gene BMRF1 or the E-GLC epitope of BMLF1 and the WQW epitope which originates from the L expressed gene BNRF1. C) Donor 3 and 8 shBILF1-LCLs were used as targets for HLA-B7 restricted effector T cells specific to the DPY epitope of the IE gene BZLF1, the RPG epitope derived from an E gene BNLF2b and the YPR epitope which originates from the L expressed gene BNRF1. All representative data are shown as fold increase in recognition of shBILF1-LCLs compared to shControl transduced LCL counterparts, following normalisation of T cell recognition (IFN-γ release) against the expression levels of the antigen from which each epitope is derived.

As shown in Figure 4A (upper graph), in marked contrast to the results observed for BNLF2a-depleted LCLs, donor 2 LCLs with reduced expression of BILF1 resulted in a 25-fold increase in recognition of the L antigen (FLD epitope of BALF4) compared to recognition of shControl-LCLs. There was a substantial, though smaller 8-fold increase in recognition of the E antigen (GLC epitope of BMLF1), and no increase in recognition of the IE antigen (YVL epitope of BRLF1). This same panel of T cells when used as effectors against Donor 3 LCLs (Figure 4A, lower graph) gave the same pattern of results, albeit a marginal 2-fold increase was observed for recognition of the IE antigen (YVL epitope of BRLF1).

A second panel of HLA-A2 restricted T cells, which included specificities towards the TLD epitope of the BMRF1 E antigen and the WQW epitope from the BNRF1 L antigen, again revealed a similar pattern of enhanced recognition of L and E epitopes (Figure 4B). Furthermore, the pattern was consistent when using our panel of HLA-B7 restricted T cells (Figure 4C). The experiments shown in Figure 4 were repeated and extended to include other donor LCLs, and summarised in Table 5. Taken together, these data show that BILF1 plays a more dominant role in interfering with antigen presentation during L stage lytic cycle (IE<<E<L) at a time when the effects of BNLF2a are diminished.

**Table 5:** Summary of fold increase in CD8 T cell recognition of EBV-antigens presented by shBILF1-LCLs compared to shControl-LCLs.

					Fold increase	
Expression phase of target antigen	Target antigen	Epitope	HLA restriction	Number of experiments*	Range	Median
IE	BRLF1	YVL	A2	10	<1–2.5	1.9
IE	BZLF1	DPY	B7	4	1.2–1.8	1.7
E	BMLF1	GLC	A2	8	7–11	9.1
E	BMRF1	TLD	A2	5	3.2–10	6
E	BNLF2b	RPG	B7	4	4.5–7	6
L	BALF4	FLD	A2	8	10–25	13.5
L	BNRF1	WQW	A2	4	5.7–16	12.2
L	BNRF1	YPR	B7	4	9–23	11.3

* more than one effector clone specific for each epitope was used where possible. In total, six different donor LCLs were used.

### Direct comparison of the effects of BNLF2a, BGLF5 and BILF1 on recognition of lytic epitopes

**Figure 5:**
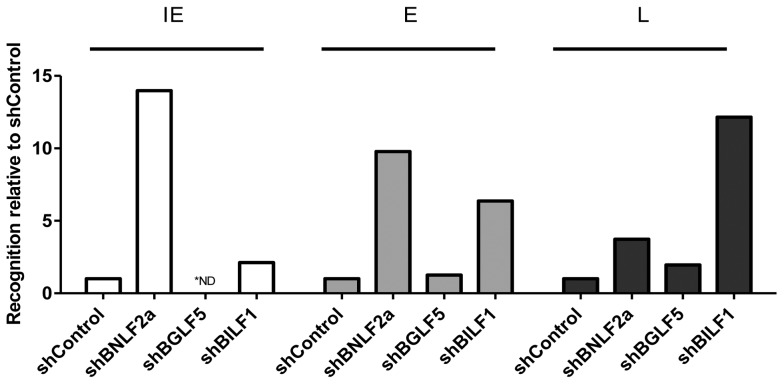
Direct comparison of the relative effects of BNLF2a, BGLF5 and BILF1 on T cell recognition of IE-YVL (BRLF1), E-GLC (BMLF1) and L-FLD (BALF4) epitopes. Recognition of epitopes presented by each knockdown and control LCL was measured simultaneously. T cell recognition (IFN-γ release) was then normalised on the expression of each appropriate target mRNA transcript. Data are shown as recognition of knockdown LCLs relative to recognition of shControl LCLs. * For one target (IE-YVL in shBGLF5) expression of target transcripts was insufficient to assay, and no T cell recognition was observed, as indicated by ND.

The data presented in Figures 2–4 and Tables 3–5 were derived from experiments where the effects of knocking down BNLF2a, BGLF5 and BILF1 on epitope recognition were assayed separately. However, in a small number of experiments, it was possible to examine in parallel the effect of knocking down expression of each of these three genes on CD8^+^ T cell recognition of IE, E and L derived epitopes. Figure 5 shows one such representative example of two replicate experiments.

The results are consistent with the general conclusions drawn from Figures 2–4 and Tables 3–5, which are: (i) that during the IE stage of lytic cycle BNLF2a plays a dominant role in interfering with antigen presentation while BILF1 contributes a small effect, (ii) at E stage lytic cycle both BILF1 and BNLF2a impair presentation, (iii) at L stage lytic cycle, BILF1 seemingly plays a dominant role, with BNLF2a contributing a small effect, and (iv) BGLF5 appears to only minimally impact on presentation throughout lytic cycle.

**Figure 6:**
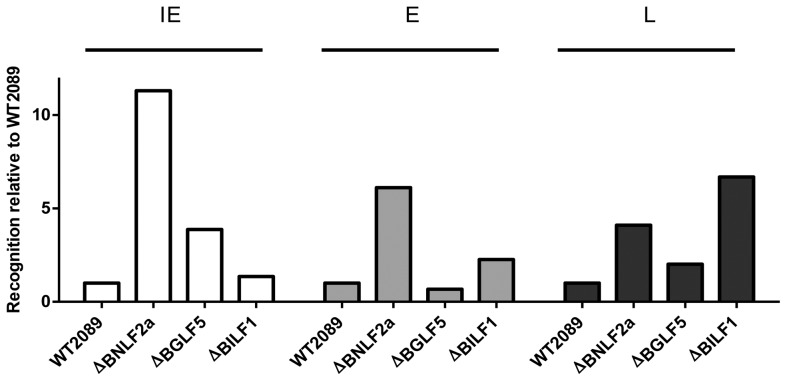
Direct comparison of the relative effects of BNLF2a, BGLF5 and BILF1 on T cell recognition of IE, E and L lytic epitopes using B-cells transformed with ΔBNLF2a, ΔBGLF5 and ΔBILF1 viruses. T cell recognition of epitopes presented by each LCL was measured simultaneously. Recognition (IFN-γ release) was normalised on the expression of each respective target mRNA transcript. Data is shown as recognition of knockout LCLs relative to recognition of WT-2089-LCLs and is the mean of two experiments using a total of two different IE-YVL (BRLF1) T cells, one E-GLC (BMLF1) and one E-TLD (BMRF1) T cell and two different L-FLD (BALF4) T cells. The complete set of individual results is presented in the Supplementary Information, Figures S6–S8.

We considered the possibility that the lack of effect of BGLF5 might possibly be due to insufficient knockdown of this gene. We therefore employed a complementary experimental approach in which we used as targets a panel of LCLs generated with rEBV in which BNLF2a, BGLF5 or BILF1 genes were knocked out. Whilst this approach was hampered by the fact that only a few LCLs demonstrated sufficient spontaneous lytic gene expression, we were able to generate sufficient data for direct comparison with the knockdown data in Figure 5. As shown in Figure 6 (and Figures S6–S8) these recombinant EBV LCLs, which completely lacked expression of BNLF2a, BGLF5 or BILF1, revealed the same pattern of results as was obtained with shRNA-mediated knockdown LCLs.

### The expression kinetics of EBV immune evasion genes partially explains their relative contribution to evasion of CD8^+^ T cell recognition

One factor that might contribute to the differential effects of BNLF2a, BGLF5 and BILF1 on CD8^+^T cell recognition of IE, E, and L antigens is the initial kinetics of their expression during lytic cycle. To address this possibility, we analysed the expression kinetics of these genes the using the EBV infected-Akata Burkitt lymphoma cell line, in which synchronous initiation of the lytic cycle of the resident virus can be induced by ligation of the B-cell receptor.

**Figure 7:**
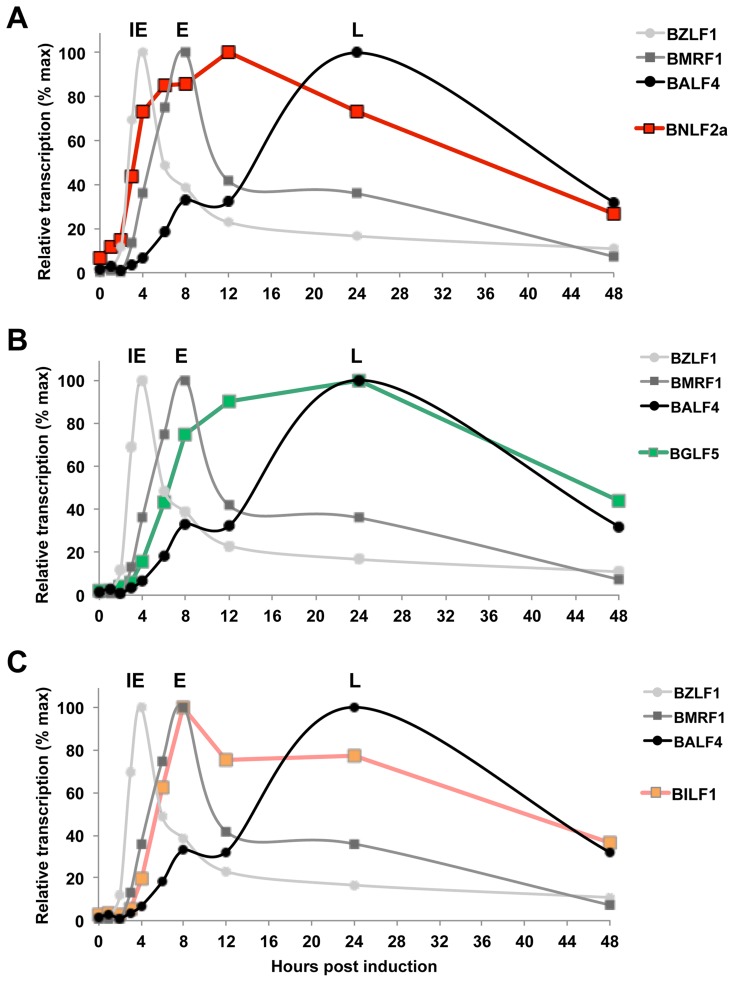
Expression kinetics of EBV lytic cycle. EBV infected cells (Akata-BL) were synchronously induced into lytic cycle by ligation of the BCR. RNA was harvested at the indicated time points and cDNA was then synthesised followed by qRT-PCR analysis to detect the expression of IE-BZLF1, E-BMRF1 and L-BALF4 (A–C). The expression of these genes is compared to expression of BNLF2a (A), BGLF5 (B) and BILF1 (C). Samples were tested in duplicate and normalised to cellular GAPDH. Data are expressed as the relative number of transcripts as percentage of the maximum for each gene.

Following induction of lytic cycle, aliquots of cells were taken at sequential time points and qRT-PCR analysis of lytic gene expression was performed. As shown in Figure 7A, BNLF2a expression is first detectable 2 h post-induction, almost coincident with the BZLF1 IE gene, and before expression of the representative E gene, BMRF1. BNLF2a expression then steadily increases and peaks 8–24 h before steadily decreasing thereafter. Thus, although BNLF2a is considered an E expressed lytic gene by virtue of its transcript being sensitive to new protein synthesis and independent of viral DNA replication, it is temporally more akin to an IE gene. Notably, the expression of BNLF2a transcript remains high, at around 73% of maximum at 24 h post-induction when maximal levels of the representative L antigen, BALF4, transcripts are expressed. However, it is known that BNLF2a protein expression is markedly diminished from 12 h post induction [19] despite the maintenance of this relatively high level of transcripts. The kinetics of expression therefore offers an explanation for why BNLF2a is most effective at interfering with antigen presentation during IE and E phase lytic cycle.

BGLF5 transcripts can be detected at the same time as BILF1, (4 h post induction), after which its expression level increases more slowly, peaking at 24 h during L-phase lytic cycle (Figure 7B). The initial expression of BILF1 is detected at around 4 h post-induction (Figure 7C), coincident with expression of BMRF1 transcripts. Having reached peak levels at 8 h, BILF1 transcripts decline slightly but are maintained at near maximal levels well into the L-phase of lytic cycle, at 24 h and beyond. This may explain why BILF1 has a subtle effect on the presentation of IE lytic epitopes and a stronger effect on L-lytic epitope presentation.

Taken together these kinetics data suggest that the roles that BNLF2a and BILF1 play in interfering with antigen presentation are at least in part a consequence of timing of their expression.

### BILF1 and BNLF2a co-operate to minimise recognition of EBV infected cells by CD8 T cells

**Figure 8:**
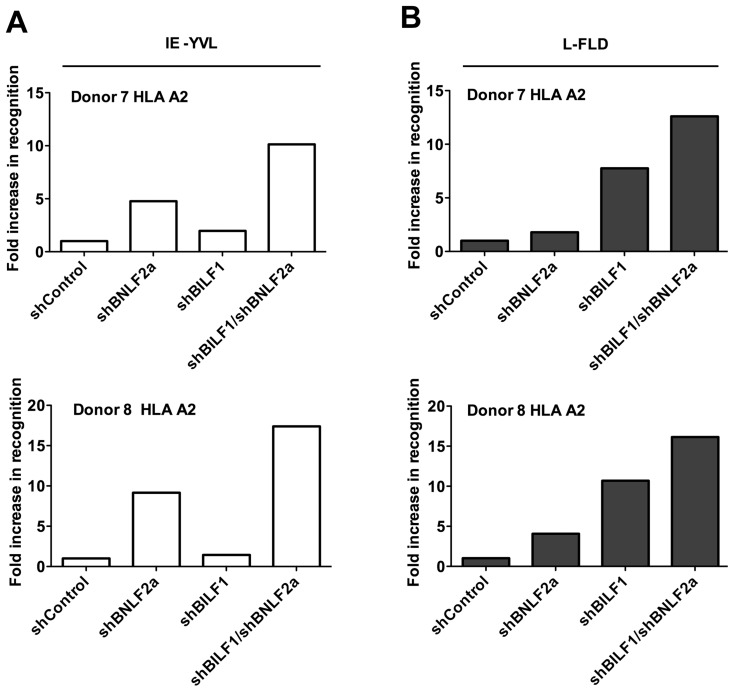
Relative recognition by IE- and L- specific, HLA-A2 restricted CD8^+^ T cell clones of LCLs lacking both BNLF2a and BILF1 expression. A) Recognition of IE-YVL presented by donors 7 and 8 LCLs was measured simultaneously. T cell recognition (IFN-γ release) was normalised on the expression of BRLF1 mRNA transcript. Data are shown as recognition of single and double knockdown LCLs relative to shControl LCLs. (B) Recognition of L-FLD presented by each donor 7 and 8 LCLs was measured simultaneously. T cell recognition (IFN-γ release) was then normalised on the expression of BRLF1 mRNA transcript. Data are shown as recognition of single and double knockdown LCLs relative to recognition of shControl LCLs.

One question arising from the preceding observations is whether there is any redundancy or co-operation between BILF1 and BNLF2a during the IE phase when individually BNLF2a is more dominant and at the L stage, when BILF1 has the strongest effect. To address this question, LCLs were transduced with both shRNA-BILF1 and shRNA-BNLF2a vectors to generate double-knockdown target LCLs. The recognition of IE (YVL from BRLF1) and L (FLD from BALF4) epitopes presented by these cells was then assessed, alongside the recognition of their single knockdown and shControl-LCL counterparts. A representative example of two repeat experiments is shown in Figure 8A, the knockdown of both BNLF2a and BILF1 expression in target cell lines increased the recognition of the IE YVL epitope 15-fold versus 9-fold for BNLF2a knockdown and 10-fold versus 5-fold for BNLF2a knockdown in two different donor LCLs. In both cases there was a minimal effect from the knockdown of BILF1 expression only. The increase in recognition using dual knockdown however suggests a level of synergy or cooperation between these two immune evasion proteins at the IE stage of lytic cycle.

Representative results obtained using L-FLD antigen specific effector CD8 T cells on the same two donor LCLs (Figure 8B), showed a clear increase in L-FLD recognition of dual knockdown LCLs compared to BILF1 only knockdown LCLs (12.5-fold versus 7.5-fold, and 16-fold versus 10.5-fold). This reproducible increase in recognition suggests that BILF1 and BNLF2a cooperate with each other at L-phase as well as at IE-phase of lytic cycle.

## Discussion

These experiments reveal that the relative contribution of BNLF2a, BGLF5 and BILF1 towards interference with antigen presentation differs during the three different phases of lytic cycle. BNLF2a has a more dominant role during the IE- and E-phases of lytic cycle, with its effect decreasing as lytic cycle progresses (IE>E>>L). Conversely, BILF1 becomes more dominant as lytic cycle progresses (IE<E<<L), coincident with declining effects of BNLF2a. Unexpectedly, our experiments revealed that the effect of BGLF5 on antigen presentation is weak throughout lytic cycle, despite its expression and host shut-off function during the E and especially L stages. Experiments using recombinant EBV deleted for the BGLF5 gene also demonstrated comparatively little effect on CD8^+^ T cell recognition (Figure 6), ruling out the theoretical possibility that the results in Fig. 3 and Table 4 were due to insufficient knockdown of BGLF5 by the shRNA approach.

The minimal effect of BGLF5 on epitope presentation is surprising, given that the ectopic expression of BGLF5 can result in a decrease in MHC class I surface expression and to significant impairment of EBV epitope recognition [17], [23]. A possible explanation for this observation is that removal of BGLF5 might cause a counteracting upregulation of other immune evasion genes. This seems not to be the case in respect of BNLF2a or BILF1 (Figure S9) although we cannot rule out the possibility that another as yet unidentified immune evasion gene is so affected. On the available evidence, we are drawn to conclude that the global down regulation of host mRNAs by BGLF5 confers little protection from CD8^+^ T cell recognition in the context of EBV infection of normal B lymphocytes. Since as few as 10 MHC/peptide molecules on the cell surface may be sufficient for recognition by CD8^+^ T cells [11], LCLs would appear to express a huge excess of MHC class I molecules. A BGLF5-mediated partial reduction in the availability of newly synthesised HLA class I molecules might therefore be inconsequential in comparison to the effects of BNLF2a and BILF1 on the available MHC class I/peptide complexes at the cell surface. The main function of BGLF5, therefore, most likely involves the generation and processing of linear viral genomes [26] rather than to protect virus-producing cells from CD8^+^ immune T cells.

The minimal immune evasion effect of BGLF5 contrasts notably with HSV, where silencing of the virion host shut-off (*vhs*) gene results in an increase in recognition by virus specific CD8^+^ T cells [27]. Why EBV (a γ-herpesvirus) and HSV (an α-herpesvirus) differ in this respect is unclear, but could be influenced by the different host cell tropism, differences in duration of lytic cycle, and differences in the molecular mechanisms of host-shut off. With regards to this final point, it will be of interest to know whether the host shut-off protein of the only other human γ-herpesvirus, Kaposi sarcoma-associated herpesvirus (KSHV), impacts on antigen presentation in the context of KSHV lytic cycle. The molecular mechanism of the KSHV SOX protein is more similar to EBV BGLF5 than to HSV vhs [18], [22], [28]. It should be noted that β-herpesviruses (such as HCMV) do not contain a host shut-off gene, so this function is clearly not a conserved and essential mechanism for herpesvirus modulation of the MHC class I antigen processing pathway.

Whilst the different kinetics of initiation of BNLF2a and BILF1 expression (Figure 7) and the subsequent posttranslational downregulation of BNLF2a protein [19] may account for their phase-specific immune-evasion functions, they might also be predicted to limit the possibilities for co-operation at the IE and L-phases of lytic cycle. Nevertheless, we did observe such cooperation. This may be because there is a window of about 6–24 h after lytic cycle entry when BNLF2a and BILF1 are co-expressed along with IE, E and L-phase antigens. Another factor to consider is that whilst both BNLF2a and BILF1 respectively can impair the generation of MHC/peptide complexes and their transport to the cell surface, BILF1 can also target pre-existing surface MHC-I/peptide complexes for degradation [16], [24]. Consequently, those MHC/peptide complexes (be they IE, E, or L antigen-derived) that survive initial evasion mechanisms to reach the cell surface, will continue to be targeted by BILF1 even after the reduction of BNLF2a protein.

That multiple viral evasion genes should demonstrate cooperation is not unexpected; indeed such cooperation is well-documented for the β-herpesvirus, CMV [7], [15], [29]. Cooperation between multiple evasion genes provides an evolutionary advantage to the virus. In addition to a generally greater efficiency of evasion, it also allows the virus to cope with peptides presented by different MHC class I allotypes. For example, EBV BILF1 only marginally affects presentation through HLA-C alleles [25], whereas BNLF2a will target all TAP-dependent peptides. This parallels the resistance of HLA-C to US2 and US11 of HCMV [30] and the targeting of TAP by HCMV US6 [31]–[33]. However, our present study highlights an additional feature of cooperation, which is to maximally impair presentation through different phases of lytic cycle. This may be particularly important for γ-herpesviruses, such as EBV, which have a relatively prolonged lytic cycle, and less important for α-herpesviruses, such as HSV, where lytic virus replication is more rapid.

Our data beg the question as to why EBV would downregulate the expression of BNLF2a at the L-phase of lytic cycle, when it is clearly such a potent immune evasion mechanism? One possibility is that excessive immune-evasion mechanisms contributing to the down regulation MHC class I levels could leave cells too vulnerable to NK cell destruction [34], [35]. In this scenario, it is envisaged that controlled expression of BNLF2a and BILF1 is perhaps an eloquent trait of EBV, in order to maximise protection from CD8^+^ T cell recognition, while minimising NK cell induced destruction. In this context it may be relevant that BILF1 preferentially targets HLA-A and HLA-B MHC class I molecules, while it does not down regulate the surface expression of HLA-C molecules which would act as NK inhibitory ligands [25]. It should also be noted that many immune-modulating viral genes have other functions relevant to the efficient replication of virus. In the case of EBV, BILF1 is a G-protein-coupled receptor whose signalling functions are dispensable for evasion from CD8^+^ T cell recognition [18], [24], [36], [37]. To date, no function for BNLF2a other than its inhibition of TAP has been defined, but the possibility remains that it has a second function in lytic replication for which prolonged high expression during late lytic cycle might be detrimental to the virus.

Previous studies have shown that the immune response to EBV is unique amongst the herpesviruses in that EBV-specific CD8^+^ T cell responses directed towards lytic antigens show a different pattern of immunodominance [14]. These EBV-specific T cell responses are more frequently skewed towards IE-phase and some E-phase lytic antigens than L-phase antigens [14]. This is likely to be due in part to the role that EBV infected B lymphocytes play in the stimulation of EBV-specific T cells. Although EBV lytic cycle can occur in both epithelial cells and in B lymphocytes [9], it appears from observations on X-linked lymphoproliferative disease (XLP) patients or heterozygous carriers of this disease that infected B cells drive stimulation of CD8^+^ T cell responses to EBV lytic cycle antigens [38]–[40]. Importantly, IE and E specific T cell responses are less able to recognise and lyse EBV infected cells that are at the L phase of lytic cycle, i.e. expressing VCA, despite continued expression of the IE and E target antigens (Pudney et al, [14]; and Figure S10). It is therefore likely that CD8^+^ T cells *in vivo* will be very inefficient at preventing the spread of EBV virus from infected cells that have already entered late lytic cycle. Extrapolating from the kinetics of lytic cycle induction in the Akata cell model (Figure 7) there would be a rather small window of perhaps 4–6 hours during E lytic cycle in which lytic EBV infected cells can be recognised and lysed in order to prevent the subsequent release of virus particles. Thereafter, the cells may produce virus for several days [12] unthreatened by immune T cell responses.

**Figure 9:**
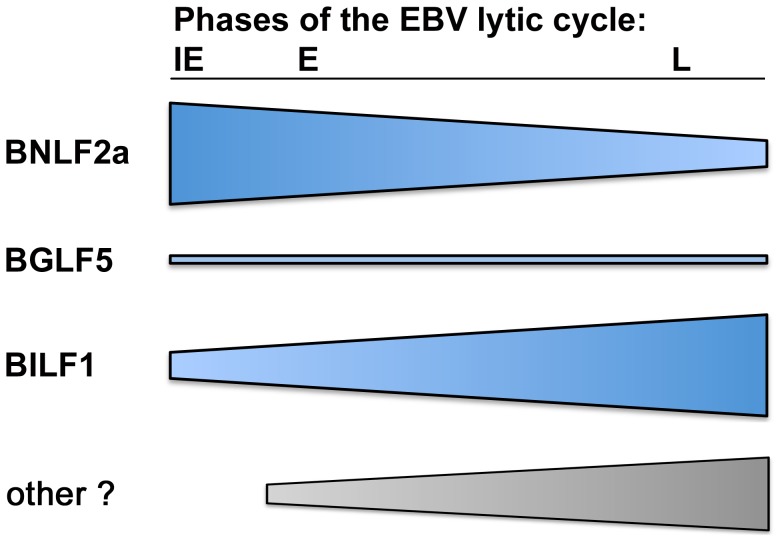
The relative roles of BNLF2a, BILF1 and BGLF5 in interfering with antigen presentation as lytic cycle progresses. Diagram showing the strength of each immune evasion gene function at all stages of lytic cycle. BNLF2a is more potent at the IE time point and its effect diminishes as lytic cycle progresses. The potency of BILF1 increases as lytic cycle progresses. BGLF5 plays a minimal role throughout.

Understanding that endogenous antigen presentation in lytically infected B cells is the predominant source of stimulation for CD8^+^ T cell responses to lytic cycle antigens, as opposed to cross-presentation via dendritic cells as is common for other herpesviruses such CMV [41]–[43], places greater importance on the role of the phase-specific interference of antigen presentation identified in the present work. In this context our new data implicate a significant contribution of BILF1 to the pattern of immunodominance that is seen for EBV. However, whilst our data demonstrate that BILF1 and BNLF2a cooperate to afford evasion across all three phases of lytic cycle, they do not obviously suggest that BILF1 is substantially more potent at the L-phase than is BNLF2a at the IE-phase. Although such differences in potency could be masked by the experimental design of our experiments, we consider it likely that there is yet to be identified one (or more) additional immune evasion gene that preferentially modulates recognition by CD8^+^ T cells specific for L-stage antigens. From the data presented in this study, a model is proposed (Figure 9).

In conclusion, the present study identifies lytic cycle phase-specific effects of viral immune evasion genes targeting the MHC class I antigen processing pathway which provides mechanistic insight into the pattern of immunodominance of EBV lytic antigen specific CD8^+^ T cell responses that sets EBV apart from other herpesvirus infections.

## Materials and Methods

### Ethics statement

Written, informed consent was given by all donors for the collection and use of blood samples, and all experiments were approved by the West Midlands (Black Country) Research Ethics Committee (07/Q2702/24).

### Production of shRNA-lentivirus

For the generation of replication-defective lentivirus, the packaging cell line FT293 (Invitrogen) was co-transfected, using lipofectamine 2000 (Invitrogen), with lentiviral vector plasmids (shBILF1-YFP, shBNLF2a-CFP or shBGLF5-FP635), (Sigma-Aldrich; Table 1), the envelope plasmid-pMD2G and the packaging plasmid-psPAx2 (Invitrogen). Supernatants containing virus were harvested 72 hours after transfection, filtered through a 0.22 µm pore and subsequently concentrated by centrifugation prior to infection of target EBV-transformed lymphoblastoid cell lines (LCLs).

### Generation of shRNA transduced target cell lines

LCLs were generated by transforming B-lymphocytes from donors of known HLA type with the B95.8 strain of EBV as previously described [19]. B95.8 transformed LCL cultures were selected on the basis of containing at least 1% of cells expressing BZLF1 protein detected by intracellular staining and flow cytometry. All LCLs were maintained in standard media (RPMI-1640 with 10% FCS). Replicate cultures of LCLs were transduced in parallel with the appropriate knockdown and control shRNA-lentiviruses (Table 1). Transduced cultures were maintained and expanded in standard media plus 1 µg/ml puromycin where necessary. For target cell lines that were more than 70% transduced after expansion, cells were used immediately in T cell recognition assays. For transduced lines in which less than 70% were transduced, enrichment was achieved by sorting on the expression of CFP, YFP or FP635 using Cytomation MoFlo fluorescence activated cell sorting. Cells were then re-cultured and maintained in standard media, until numbers were sufficient for use in T cell recognition assays.

### Generation of recombinant EBV gene knockout transformed LCLs

Wild-type recombinant EBV based on the B95.8 genome, 2089, and null recombinants for BNLF2a, BGLF5, or BILF1, or BZLF1 have been described elsewhere [19], [26], [44], [45]. The 2089, ΔBGLF5, ΔBILF1 and ΔBZLF1 recombinant viruses were kindly provided by Henri Jacques Delecluse and Regina Feederle, Heidelberg. LCLs carrying these recombinant EBVs were generated by transforming B lymphocytes from donors of known HLA type with the B95.8 strain of EBV as previously described [19].

### CD8 T cell recognition assays

CD8^+^ T cell clones were generated as previously described [14], [46] using limiting dilution or IFN-γ capture T cell cloning. All novel HLA-B7 restricted T cell clones were generated using limiting dilution cloning while HLA-A2 restricted effector clones were from IFN-γ capture and limiting dilution T cell cloning. The clones used in this study are shown in Table 2. CD8^+^ T cell recognition of lytic epitopes presented by shRNA-transduced LCLs was measured using a standard IFN-γ ELISA assay as previously described [47]. Briefly, triplicate aliquots of 10^5^ target LCLs were incubated with 10^4^ effector T cells for 18 h in standard media. To measure T cell recognition of the target cells, 50 µl of the supernatant from each well was assayed for IFN-γ.

### Quantitative real-time reverse transcription PCR (qRT-PCR)

Total RNA was extracted from 0.5×10^6^ to 10^6^ cells using RNeasy kit (Nugen) followed by Turbo DNA-free (Applied biosystems) treatment to remove any contaminating DNA. A 500 ng sample of RNA was reverse transcribed into cDNA using qScript cDNA supermix, as per manufacturer's protocol (Quanta biosciences). Quantitative-PCR was then performed using specific EBV lytic gene primers (Alta Bioscience) and probes (Eurogentec) (Table S1). Expression normalised to GAPDH expression and the data displayed as relative to expression in shNon-target LCLs, or relative to the maximal level of transcript for each gene.

### Method of normalisation of CD8^+^ T cell recognition experiments

T cell recognition assays relied upon target LCLs spontaneously entering lytic cycle replication, the efficiency of which varies between lines and within lines over time. Since this directly impacts the level of antigen available for presentation, and therefore CD8^+^ T cell recognition, it was important to measure the level of target antigen expression in each cell line in every experiment. As peptides for presentation to T cells are generally considered to originate predominantly from the products of defective translation (DRiPs) rather than through degradation of mature protein [48]–[50], we measured the level of mRNA transcript of each antigen to which our T cells were specific. This allowed us to normalise the amount of IFN-γ release (T cell recognition) against target antigen expression. For example, for a CD8^+^ T cell which recognises the YVL epitope, derived from the lytic antigen BRLF1, if the mRNA level of BRLF1 in the reference target line (shControl LCLs) was *y*, and in T cell recognition (IFN-γ release) was *x*, then the amount of IFN-γ released by YVL specific T cells incubated with the reference line was adjusted (normalised) by dividing *x* by *y*. This was performed on all lines which enabled us to express the recognition data as fold increase in epitope recognition of knockdown LCLs as a ratio of recognition of shControl LCLs. The validity of this experimental approach was demonstrated by the direct correlation between the level of target antigen-mRNA and CD8^+^ T cell recognition, as shown in Figure S1. Thus, by measuring the mRNA-expression level of specific target antigens we can accurately account for differences in the amount of lytic cycle in individual LCL target cell lines on the day of assay. Examples of raw T cell recognition and mRNA expression data alongside the subsequent normalised data are shown in Figures S2–S5.

### Western blot analysis

Western blotting was performed as described previously [51]. Briefly, total cell lysates were prepared in reducing sample buffer (2% sodium dodecyl sulphate (SDS), 72.5 mM Tris-HCl pH 6.8, 10% glycerol, 02.M sodium 2-mercaptoethanesulfonate, 0.002% bromophenol blue), sonicated and heated to 100°C for 5 min. Solubilised proteins equivalent to 2×10^5^ cells/20 µl sample were separated by SDS-polyacrylamide gel electrophoresis on 4–12% Bis-Tris NuPage mini-gels with morpholinepropanesulfonic acid running buffer (Invitrogen), then transferred to polyvinylidene difluoride membranes. Specific proteins were detected by incubating membranes with primary antibodies at 4°C overnight. Rabbit anti-BGLF5 serum [52] was diluted 1/6,000, clone 5B9 rat anti-BNLF2a [19] culture supernatant was used at a dilution of 1/100, clone BZ1 purified mouse anti-BZLF1 [53] and goat anti-calregulin (sc6467; Santa Cruz Biotechnology) were used at 1 µg/ml. Primary antibody binding was detected by incubation with appropriate alkaline phosphatase conjugated secondary antibody and subsequently developed using CDP-star detection kit (Applied Biosystems).

### Synchronous induction of lytic cycle in the Akata-BL line

The reactivation of Akata-BL cells into lytic cycle was performed by cross-linking surface IgG molecules as previously described [54]. Cells were then harvested at the indicated time points for qRT-PCR analysis.

## Supporting Information

Figure S1 **Correlation between mRNA antigen expression and CD8^+^ T cell recognition.** A B95.8-LCL line was selected in which 5% of the cells were expressing the lytic switch protein BZLF1 (detected via intracellular staining with BZ.1 monoclonal antibody). These lytic cells were then serially diluted with tightly-latent ΔBZLF1-LCLs, so that the proportion of lytic cell line ranged from 100% to 0%. These cell mixes were then used as targets for a GLC-specific CD8^+^ T cell clone in a T cell recognition assay. Recognition is shown as percentage IFN-γ release, where 100% release is that seen in undiluted lytic B95.8 LCLs (5% BZLF1 positive). An aliquot of these cell mixes was also taken to extract RNA and carry out qRT-PCR analysis to detect the level of BMLF1 mRNA. This is shown as % of BMLF1, where 100% is taken as the level of BMLF1 in the lytic B95.8-LCLs before dilution with ΔBZLF1-LCLs cells.
(TIF)

Figure S2 **Recognition of donor 3 shBNLF2a-LCLs.** (A) Recognition of donor 3 LCLs by a IE-YVL, E-GLC and L-FLD specific CD8^+^ T cell clones. Recognition is shown as IFN-γ (pg/ml) release by T cells. Maximal experimental recognition is indicated by recognition of peptide-sensitised ΔBZLF1 LCLS. (B) Levels of IE-BRLF1, E-BMLF1 and L-BALF4 mRNA transcripts in the target LCLs used in A. (C) Recognition of donor 3 shBNLF2a-LCLs relative to donor 3 shControl-LCLs, after normalisation of IFN-γ release against transcript level.
(TIF)

Figure S3 **Recognition of donor 4 shBNLF2a-LCLs.** (A) Recognition of donor 4 LCLs by IE-YVL, E-GLC and L-FLD specific CD8^+^ T cell clones. Recognition is shown as IFN-γ (pg/ml) release. Maximal experimental recognition is indicated by recognition of peptide-sensitised ΔBZLF1 LCLS. (B) Levels of IE-BRLF1, E-BMLF1and L-BALF4 mRNA transcripts in the target LCLs used in A. (C) Recognition of donor 4 shBNLF2a-LCLs relative to donor 4 shControl-LCLs, after normalisation of IFN-γ release against target transcript levels.
(TIF)

Figure S4 **Recognition of donor 5 shBNLF2a-LCLs.** (A) Recognition of donor 5 LCLs by IE-YVL, E-TLD and L-WQW specific CD8^+^ T cell clones. Recognition is shown as IFN-γ (pg/ml) release. Maximal experimental recognition is indicated by recognition of peptide-sensitised ΔBZLF1 LCLS. (B) Level s of IE-BRLF1, E-BMRF1 and L-BNRF1 mRNA transcripts in the target LCLs used in A. (C) Recognition of donor 5 shBNLF2a-LCLs relative to donor 5 shControl-LCLs, after normalisation of IFN-γ release against target transcript levels.
(TIF)

Figure S5 **Recognition of donor 6 shBNLF2a-LCLs.** A) Recognition of donor 6 LCLs by IE-YVL, E-TLD and L-WQW specific CD8^+^ T cell clones. Recognition is shown as IFN-γ (pg/ml) release. Maximal experimental recognition is indicated by recognition of peptide-sensitised ΔBZLF1 LCLS. (B) Levels of IE-BRLF1, E-BMRF1and L-BNRF1 mRNA transcripts in the target LCLs used in A. (C) Recognition of donor 6 shBNLF2a-LCLs relative to donor 6 shControl-LCLs, after normalisation of IFN-γ release against target transcript levels.
(TIF)

Figure S6 **Recognition of donor 7 LCLs by IE-YVL specific CD8^+^ T cell clones.** A) Recognition of KO-LCLs by two YVL-specific clones is shown as IFN-γ (pg/ml) release. Maximal recognition is indicated by recognition of peptide-sensitised ΔBZLF1-LCLs. B) mRNA levels of BRLF1 in target LCLs. C) Recognition of LCLs relative to WT2089-LCLs, after normalisation of IFN-γ release against transcript levels.
(JPG)

Figure S7 **Recognition of donor 7 KO-LCLs by E-GLC and -TLD specific CD8^+^ T cell clones.** A) Recognition of KO-LCLs shown as IFN-γ (pg/ml) release. Maximal recognition is indicated by recognition of peptide-sensitised ΔBZLF1-LCLs. B) mRNA levels of corresponding BMLF1 and BMRF1 in target LCLs. C) Recognition of LCLs relative to WT2089-LCLs, after normalisation of IFN-γ release against transcript levels.
(JPG)

Figure S8 **Recognition of donor 7 KO-LCLs by two L-FLD specific CD8^+^ T cell clones.** A) Recognition of KO-LCLs shown as IFN-γ (pg/ml) release. Maximal recognition is indicated by recognition of peptide-sensitised ΔBZLF1-LCLs. B) mRNA levels of BALF4 in target LCLs. C) Recognition of LCLs relative to WT2089-LCLs, after normalisation of IFN- γ release against transcript levels.
(JPG)

Figure S9 **The effect of BGLF5 knockout on lytic gene and protein expression.** WT2089- and counterpart ΔBGLF5 knockout-LCLs were transduced with either a pRTS-CD2-control or pRTS-CD2-BZLF1 vector. This vector carries a bidirectional doxycycline (Dox) regulatable promoter, BI-Tet, which drives the expression of BZLF1, which is able to induce lytic cycle, together with a non-functional neuronal growth factor receptor (NGFR) and green fluorescent protein (GFP) as a markers of Dox induced expression. WT2089- and ΔBGLF5-LCLs transfected with pRTS-CD2-BZLF1 or pRTS-CD2-control vector were treated for 12 hrs with Dox before selecting for induced plasmid containing cells using MACSelect LNGFR MicroBeads. (A) In one experiment, RNA was extracted from the selected cells and used to generate cDNA in order to analyse the expression of a panel of lytic cycle genes using qRT-PCR. This panel included 2 IE genes, 7 E-genes which included the immune evasion genes BNLF2a, BGLF5 and BILF1 and 3 L genes. Plotting the expression levels of each of these genes in lytically induced WT2089-LCLs (WT2089+BZLF1) alongside lytically induced ΔBGLF5-LCLs (ΔBGLF5+BZLF1) allows us to directly compare the impact of BGLF5 knockout on the expression of lytic genes. Variation in BZLF1 expression, and lytic cycle induction, between WT2089+BZLF1 and ΔBGLF5+BZLF1 LCLs were compensated by displaying of all genes relative to the expression of BRLF1 in that cell. (B) In a separate experiment, selected WT2089-control and −BZLF1 (lane 1 and 2 respectively) and ΔBGLF5-control and −BZLF1 (lane 3 and 4) transfected LCLs were also analysed by SDS-PAGE and immunoblotting with antibodies specific for the lytic cycle proteins BZLF1, BNLF2a, BHRF1, BMLF1, and BALF2, with calregulin as a loading control.
(JPG)

Figure S10 **VCA^+^ lytically infected cells are resistant to E-antigen specific effector T cells.** HLA A2 positive LCLs containing around 2% cells spontaneously in lytic cycle were co-cultured with or without GLC T cells (A2 restricted and BMLF1 specific cytotoxic CD8^+^ effector clone) at a ratio of 1∶1 for 16 hr. The total cell population was then harvested and stained with anti-CD19 to identify the LCL B cells, then fixed and permeabilized, and lytic LCLs were identified through intracellular for BZLF1 and VCA. The percentage of BZLF1^+^ B cells in the culture without GLC T cells was set as 100%, and the number of BZLF1^+^ or VCA^+^ lytic LCLs remaining following incubation with GLC-specific T cells is shown relative to this. The data show a 60% reduction in the number of BZLF1^+^ B cells following co-culture with GLC T cells, but no significant depletion of VCA^+^ B cells.
(TIF)

Table S1 **EBV lytic gene primers and probes used for qRT-PCR.**
(DOCX)

## References

[1] Zuckerman RA, Limaye AP (2013) Varicella zoster virus (VZV) and herpes simplex virus (HSV) in solid organ transplant patients. Am J Transplant 13 Suppl 3: 55–66; quiz 66. 10.1111/ajt.12003 23347214

[2] Hebart H, Einsele H (2004) Clinical aspects of CMV infection after stem cell transplantation. Hum Immunol 65: 432–436. 15172442 10.1016/j.humimm.2004.02.022

[3] Zerr DM, Corey L, Kim HW, Huang ML, Nguy L, et al (2005) Clinical outcomes of human herpesvirus 6 reactivation after hematopoietic stem cell transplantation. Clin Infect Dis 40: 932–940. 15824982 10.1086/428060

[4] Gottschalk S, Rooney CM, Heslop HE (2005) Post-transplant lymphoproliferative disorders. Annu Rev Med 56: 29–44. 15660500 10.1146/annurev.med.56.082103.104727

[5] Gerdemann U, Keukens L, Keirnan JM, Katari UL, Nguyen CT, et al (2013) Immunotherapeutic strategies to prevent and treat human herpesvirus 6 reactivation after allogeneic stem cell transplantation. Blood 121: 207–218. 23152545 10.1182/blood-2012-05-430413 PMC3709638

[6] Leen AM, Myers GD, Sili U, Huls MH, Weiss H, et al (2006) Monoculture-derived T lymphocytes specific for multiple viruses expand and produce clinically relevant effects in immunocompromised individuals. Nat Med 12: 1160–1166. 16998485 10.1038/nm1475

[7] Hislop AD, Taylor GS, Sauce D, Rickinson AB (2007) Cellular responses to viral infection in humans: lessons from Epstein-Barr virus. Annu Rev Immunol 25: 587–617. 17378764 10.1146/annurev.immunol.25.022106.141553

[8] Starzl TE, Nalesnik MA, Porter KA, Ho M, Iwatsuki S, et al (1984) Reversibility of lymphomas and lymphoproliferative lesions developing under cyclosporin-steroid therapy. Lancet (i) 583–587. 10.1016/s0140-6736(84)90994-2 PMC2987704 6142304

[9] Rickinson AB, Kieff E (2007) Epstein-Barr virus. In: Knipe DM, Howley PM, editors. Fields Virology, 5th Edition. Philadelphia: Lippincott, Williams & Wilkins. pp. 2655–2700.

[10] Rowe M, Kelly GL, Bell AI, Rickinson AB (2009) Burkitt's lymphoma: The Rosetta Stone deciphering Epstein-Barr virus biology. Semin Cancer Biol 19: 377–388. 19619657 10.1016/j.semcancer.2009.07.004 PMC3764430

[11] Hadinoto V, Shapiro M, Sun CC, Thorley-Lawson DA (2009) The dynamics of EBV shedding implicate a central role for epithelial cells in amplifying viral output. PLoS Pathog 5: 3. 10.1371/journal.ppat.1000496 PMC2698984 19578433

[12] Ressing ME, Keating SE, van Leeuwen D, Koppers-Lalic D, Pappworth IY, et al (2005) Impaired Transporter Associated with Antigen Processing-dependent peptide transport during productive EBV Infection. J Immunol 174: 6829–6838. 15905524 10.4049/jimmunol.174.11.6829

[13] Keating S, Prince S, Jones M, Rowe M (2002) The lytic cycle of Epstein-Barr virus is associated with decreased expression of cell surface major histocompatibility complex class I and class II molecules. J Virol 76: 8179–8188. 12134023 10.1128/JVI.76.16.8179-8188.2002 PMC155144

[14] Pudney VA, Leese AM, Rickinson AB, Hislop AD (2005) CD8+ immunodominance among Epstein-Barr virus lytic cycle antigens directly reflects the efficiency of antigen presentation in lytically infected cells. J Exp Med 201: 349–360. 15684323 10.1084/jem.20041542 PMC2213038

[15] Hislop AD, Ressing ME, van Leeuwen D, Pudney VA, Horst D, et al (2007) A CD8^+^ T cell immune evasion protein specific to Epstein-Barr virus and its close relatives in Old World primates. J Exp Med 204: 1863–1873. 17620360 10.1084/jem.20070256 PMC2118677

[16] Zuo J, Currin A, Griffin BD, Shannon-Lowe C, Thomas WA, et al (2009) The Epstein-Barr virus G-protein-coupled receptor contributes to immune evasion by targeting MHC class I molecules for degradation. PLoS Pathog 5: 2. 10.1371/journal.ppat.1000255 PMC2603334 19119421

[17] Rowe M, Glaunsinger B, van Leeuwen D, Zuo J, Sweetman D, et al (2007) Host shutoff during productive Epstein-Barr virus infection is mediated by BGLF5 and may contribute to immune evasion. Proc Natl Acad Sci U S A 104: 3366–3371. 17360652 10.1073/pnas.0611128104 PMC1805610

[18] Rowe M, Zuo J (2010) Immune responses to Epstein-Barr virus: molecular interactions in the virus evasion of CD8+ T cell immunity. Microbes Infect 12: 173–181. 20004735 10.1016/j.micinf.2009.12.001 PMC2832755

[19] Croft NP, Shannon-Lowe C, Bell AI, Horst D, Kremmer E, et al (2009) Stage-specific inhibition of MHC class I presentation by the Epstein-Barr virus BNLF2a protein during virus lytic cycle. PLoS Pathog 5: e1000490. 19557156 10.1371/journal.ppat.1000490 PMC2695766

[20] Horst D, Favaloro V, Vilardi F, van Leeuwen HC, Garstka MA, et al (2011) EBV protein BNLF2a exploits host tail-anchored protein integration machinery to inhibit TAP. J Immunol 186: 3594–3605. 21296983 10.4049/jimmunol.1002656

[21] Horst D, van Leeuwen D, Croft NP, Garstka MA, Hislop AD, et al (2009) Specific Targeting of the EBV Lytic Phase Protein BNLF2a to the Transporter Associated with Antigen Processing Results in Impairment of HLA Class I-Restricted Antigen Presentation. J Immunol 182: 2313–2324. 19201886 10.4049/jimmunol.0803218

[22] Buisson M, Geoui T, Flot D, Tarbouriech N, Ressing ME, et al (2009) A bridge crosses the active-site canyon of the Epstein-Barr virus nuclease with DNase and RNase activities. J Mol Biol 391: 717–728. 19538972 10.1016/j.jmb.2009.06.034

[23] Zuo J, Thomas W, van Leeuwen D, Middeldorp JM, Wiertz EJ, et al (2008) The DNase of gammaherpesviruses impairs recognition by virus-specific CD8+ T cells through an additional host shutoff function. J Virol 82: 2385–2393. 18094150 10.1128/JVI.01946-07 PMC2258936

[24] Zuo J, Quinn LL, Tamblyn J, Thomas WA, Feederle R, et al (2011) The Epstein-Barr virus-encoded BILF1 protein modulates immune recognition of endogenously processed antigen by targeting major histocompatibility complex class I molecules trafficking on both the exocytic and endocytic pathways. J Virol 85: 1604–1614. 21123379 10.1128/JVI.01608-10 PMC3028889

[25] Griffin BD, Gram AM, Mulder A, Van Leeuwen D, Claas FH, et al (2013) EBV BILF1 evolved to downregulate cell surface display of a wide range of HLA class I molecules through their cytoplasmic tail. J Immunol 190: 1672–1684. 23315076 10.4049/jimmunol.1102462 PMC3565383

[26] Feederle R, Bannert H, Lips H, Muller-Lantzsch N, Delecluse HJ (2009) The Epstein-Barr virus alkaline exonuclease BGLF5 serves pleiotropic functions in virus replication. J Virol 83: 4952–4962. 19264771 10.1128/JVI.00170-09 PMC2682060

[27] Tigges MA, Leng S, Johnson DC, Burke RL (1996) Human herpes simplex virus (HSV)-specific CD8+ CTL clones recognize HSV-2-infected fibroblasts after treatment with IFN-gamma or when virion host shutoff functions are disabled. J Immunol 156: 3901–3910. 8621929

[28] Garibal J, Hollville E, Bell AI, Kelly GL, Renouf B, et al (2007) Truncated form of the Epstein-Barr virus protein EBNA-LP protcts against caspase-dependent apoptosis by inhibiting protein phosphatase 2A. Journal of Virology 81: 7598–7607. 17494066 10.1128/JVI.02435-06 PMC1933342

[29] Hislop AD, Ressing ME, van Leeuwen D, Pudney VA, Horst D, et al (2007) A CD8(+) T cell immune evasion protein specific to Epstein-Barr virus and its close relatives in Old World primates. Journal of Experimental Medicine 204: 1863–1873. 17620360 10.1084/jem.20070256 PMC2118677

[30] Kelly GL, Stylianou J, Bell AI, Wei W, Rowe M, et al (2007) Three restricted forms of Epstein-Barr virus latency counteracting apoptosis in c-Myc expressing Burkitt Lymphoma cells. Blood 110: 470a–470a. 10.1073/pnas.0509988103 PMC1595454 17001014

[31] Ahn K, Gruhler A, Galocha B, Jones TR, Wiertz EJ, et al (1997) The ER-luminal domain of the HCMV glycoprotein US6 inhibits peptide translocation by TAP. Immunity 6: 613–621. 9175839 10.1016/s1074-7613(00)80349-0

[32] Hengel H, Koopmann JO, Flohr T, Muranyi W, Goulmy E, et al (1997) A viral ER-resident glycoprotein inactivates the MHC-encoded peptide transporter. Immunity 6: 623–632. 9175840 10.1016/s1074-7613(00)80350-7

[33] Hislop AD, Taylor GS, Sauce D, Rickinson AB (2007) Cellular responses to viral infection in humans: Lessons from Epstein-Barr virus. Annual Review of Immunology 25: 587–617. 10.1146/annurev.immunol.25.022106.141553 17378764

[34] Chijioke O, Azzi T, Nadal D, Munz C (2013) Innate immune responses against Epstein Barr virus infection. J Leukoc Biol 94: 1185–1190. 23812328 10.1189/jlb.0313173 PMC3828602

[35] Pappworth IY, Wang EC, Rowe M (2007) The switch from latent to productive infection in epstein-barr virus-infected B cells is associated with sensitization to NK cell killing. J Virol 81: 474–482. 17079298 10.1128/JVI.01777-06 PMC1797427

[36] Beisser PS, Verzijl D, Gruijthuijsen YK, Beuken E, Smit MJ, et al (2005) The Epstein-Barr virus BILF1 gene encodes a G protein-coupled receptor that inhibits phosphorylation of RNA-dependent protein kinase. J Virol 79: 441–449. 15596837 10.1128/JVI.79.1.441-449.2005 PMC538699

[37] Paulsen SJ, Rosenkilde MM, Eugen-Olsen J, Kledal TN (2005) Epstein-Barr virus-encoded BILF1 is a constitutively active G protein-coupled receptor. J Virol 79: 536–546. 15596846 10.1128/JVI.79.1.536-546.2005 PMC538743

[38] Coffey AJ, Brooksbank RA, Brandau O, Oohashi T, Howell GR, et al (1998) Host response to EBV infection in X-linked lymphoproliferative disease results from mutations in an SH2-domain encoding gene. Nat Genet 20: 129–135. 9771704 10.1038/2424

[39] Hislop AD, Palendira U, Leese AM, Arkwright PD, Rohrlich PS, et al (2010) Impaired Epstein-Barr virus-specific CD8+ T-cell function in X-linked lymphoproliferative disease is restricted to SLAM family-positive B-cell targets. Blood 116: 3249–3257. 20644117 10.1182/blood-2009-09-238832

[40] Nichols KE, Harkin DP, Levitz S, Krainer M, Kolquist KA, et al (1998) Inactivating mutations in an SH2 domain-encoding gene in X-linked lymphoproliferative syndrome. Proc Natl Acad Sci U S A 95: 13765–13770. 9811875 10.1073/pnas.95.23.13765 PMC24894

[41] Busche A, Jirmo AC, Welten SP, Zischke J, Noack J, et al (2013) Priming of CD8+ T cells against cytomegalovirus-encoded antigens is dominated by cross-presentation. J Immunol 190: 2767–2777. 23390296 10.4049/jimmunol.1200966

[42] Munks MW, Pinto AK, Doom CM, Hill AB (2007) Viral interference with antigen presentation does not alter acute or chronic CD8 T cell immunodominance in murine cytomegalovirus infection. J Immunol 178: 7235–7241. 17513772 10.4049/jimmunol.178.11.7235

[43] Snyder CM, Allan JE, Bonnett EL, Doom CM, Hill AB (2010) Cross-presentation of a spread-defective MCMV is sufficient to prime the majority of virus-specific CD8+ T cells. PLoS One 5: 0009681. 10.1371/journal.pone.0009681 PMC2837378 20300633

[44] Feederle R, Kost M, Baumann M, Janz A, Drouet E, et al (2000) The Epstein-Barr virus lytic program is controlled by the co-operative functions of two transactivators. EMBO J 19: 3080–3089. 10856251 10.1093/emboj/19.12.3080 PMC203345

[45] Zuo J, Quinn LL, Tamblyn J, Thomas WA, Feederle R, et al (2011) The Epstein-Barr virus-encoded BILF1 protein modulates immune recognition of endogenously processed antigen by targeting MHC class I molecules trafficking on both the exocytic and endocytic pathways. J Virol 85: 1604–1614. 21123379 10.1128/JVI.01608-10 PMC3028889

[46] Abbott RJ, Quinn LL, Leese AM, Scholes HM, Pachnio A, et al (2013) CD8+ T Cell Responses to Lytic EBV Infection: Late Antigen Specificities as Subdominant Components of the Total Response. J Immunol 191: 5398–5409. 24146041 10.4049/jimmunol.1301629 PMC5580796

[47] Long HM, Haigh TA, Gudgeon NH, Leen AM, Tsang CW, et al (2005) CD4+ T-cell responses to Epstein-Barr virus (EBV) latent-cycle antigens and the recognition of EBV-transformed lymphoblastoid cell lines. J Virol 79: 4896–4907. 15795275 10.1128/JVI.79.8.4896-4907.2005 PMC1069546

[48] Blum JS, Wearsch PA, Cresswell P (2013) Pathways of antigen processing. Annu Rev Immunol 31: 443–473. 23298205 10.1146/annurev-immunol-032712-095910 PMC4026165

[49] Croft NP, Smith SA, Wong YC, Tan CT, Dudek NL, et al (2013) Kinetics of Antigen Expression and Epitope Presentation during Virus Infection. PLoS Pathogens 9: e1003129. 23382674 10.1371/journal.ppat.1003129 PMC3561264

[50] Mackay LK, Long HM, Brooks JM, Taylor GS, Leung CS, et al (2009) T cell detection of a B-cell tropic virus infection: newly-synthesised versus mature viral proteins as antigen sources for CD4 and CD8 epitope display. PLoS Pathog 5: e1000699. 20019813 10.1371/journal.ppat.1000699 PMC2788701

[51] Rowe M, Jones M (2001) Epstein-Barr virus protocols. Detection of EBV latent proteins by Western Blotting. Methods in Molecular Biology 174: 229–242. 11357649 10.1385/1-59259-227-9:229

[52] Fachiroh J, Schouten T, Hariwiyanto B, Paramita DK, Harijadi A, et al (2004) Molecular diversity of Epstein-Barr virus IgG and IgA antibody responses in nasopharyngeal carcinoma: a comparison of Indonesian, Chinese, and European subjects. J Infect Dis 190: 53–62. 15195243 10.1086/421245

[53] Young LS, Lau R, Rowe M, Niedobitek G, Packham G, et al (1991) Differentiation-associated expression of the Epstein-Barr virus BZLF1 transactivator protein in oral “hairy” leukoplakia. J Virol 65: 2868–2874. 1851858 10.1128/jvi.65.6.2868-2874.1991 PMC240913

[54] Rowe M, Lear AL, Croom-Carter D, Davies AH, Rickinson AB (1992) Three pathways of Epstein-Barr virus gene activation from EBNA1-positive latency in B lymphocytes. J Virol 66: 122–131. 1309242 10.1128/jvi.66.1.122-131.1992 PMC238267

