# Supplementary figures and images for: Cooperation between Epstein-Barr Virus Immune Evasion Proteins Spreads Protection from CD8+ T Cell Recognition across All Three Phases of the Lytic Cycle
Source: PLoS Pathog. 2014 Aug 21;10(8):e1004322. doi: 10.1371/journal.ppat.1004322 (PMC4140850; doi:10.1371/journal.ppat.1004322)

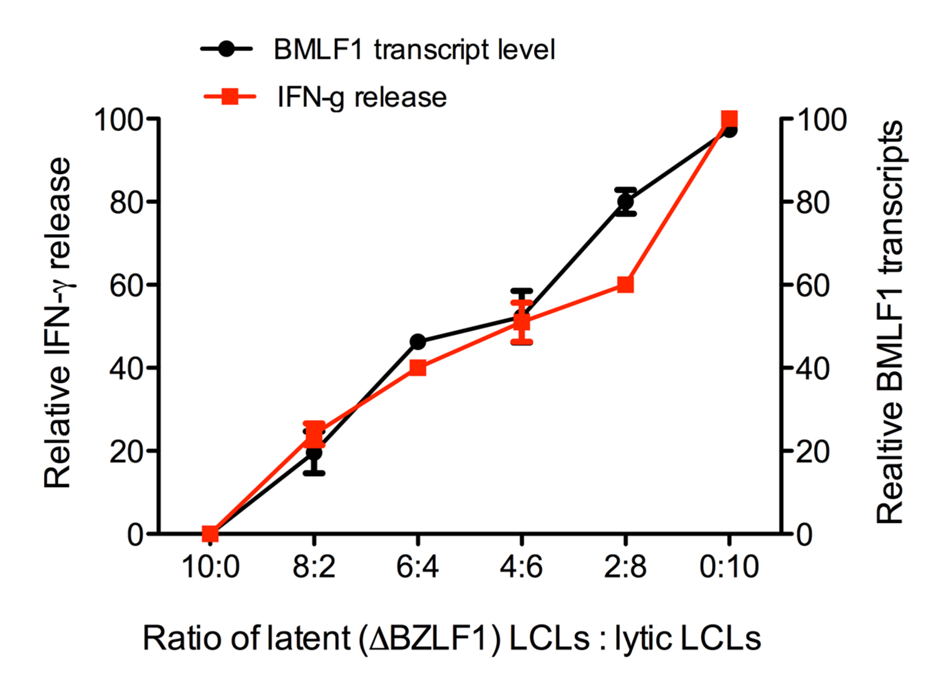

Supplement: Figure S1 — Correlation between mRNA antigen expression and CD8+ T cell recognition. A B95.8-LCL line was selected in which 5% of the cells were expressing the lytic switch protein BZLF1 (detected via intracellular staining with BZ.1 monoclonal antibody). These lytic cells were then serially diluted with tightly-latent ΔBZLF1-LCLs, so that the proportion of lytic cell line ranged from 100% to 0%. These cell mixes were then used as targets for a GLC-specific CD8+ T cell clone in a T cell recognition assay. Recognition is shown as percentage IFN-γ release, where 100% release is that seen in undiluted lytic B95.8 LCLs (5% BZLF1 positive). An aliquot of these cell mixes was also taken to extract RNA and carry out qRT-PCR analysis to detect the level of BMLF1 mRNA. This is shown as % of BMLF1, where 100% is taken as the level of BMLF1 in the lytic B95.8-LCLs before dilution with ΔBZLF1-LCLs cells. (TIF) [file ppat.1004322.s001.tif]

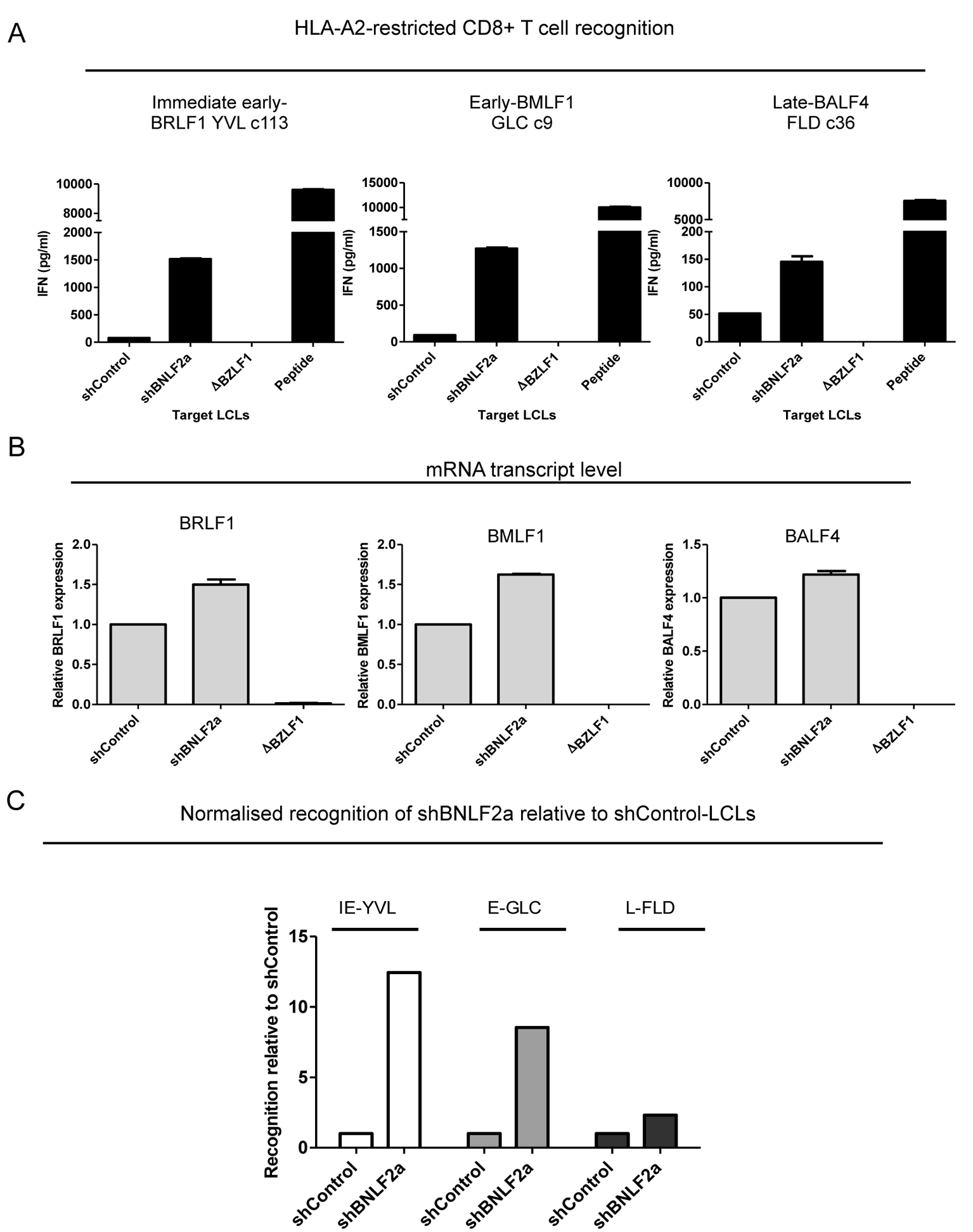

Supplement: Figure S2 — Recognition of donor 3 shBNLF2a-LCLs. (A) Recognition of donor 3 LCLs by a IE-YVL, E-GLC and L-FLD specific CD8+ T cell clones. Recognition is shown as IFN-γ (pg/ml) release by T cells. Maximal experimental recognition is indicated by recognition of peptide-sensitised ΔBZLF1 LCLS. (B) Levels of IE-BRLF1, E-BMLF1 and L-BALF4 mRNA transcripts in the target LCLs used in A. (C) Recognition of donor 3 shBNLF2a-LCLs relative to donor 3 shControl-LCLs, after normalisation of IFN-γ release against transcript level. (TIF) [file ppat.1004322.s002.tif]

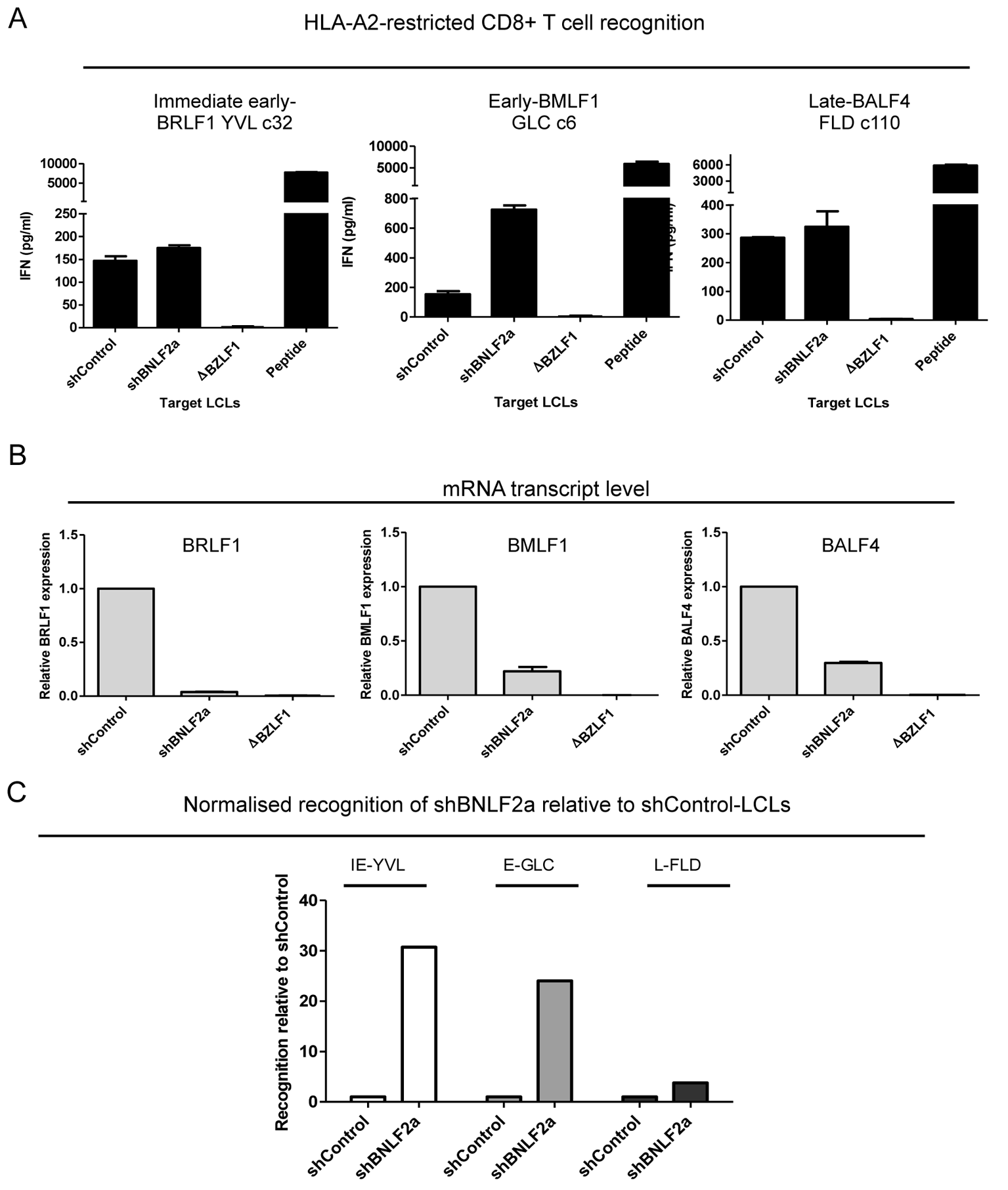

Supplement: Figure S3 — Recognition of donor 4 shBNLF2a-LCLs. (A) Recognition of donor 4 LCLs by IE-YVL, E-GLC and L-FLD specific CD8+ T cell clones. Recognition is shown as IFN-γ (pg/ml) release. Maximal experimental recognition is indicated by recognition of peptide-sensitised ΔBZLF1 LCLS. (B) Levels of IE-BRLF1, E-BMLF1and L-BALF4 mRNA transcripts in the target LCLs used in A. (C) Recognition of donor 4 shBNLF2a-LCLs relative to donor 4 shControl-LCLs, after normalisation of IFN-γ release against target transcript levels. (TIF) [file ppat.1004322.s003.tif]

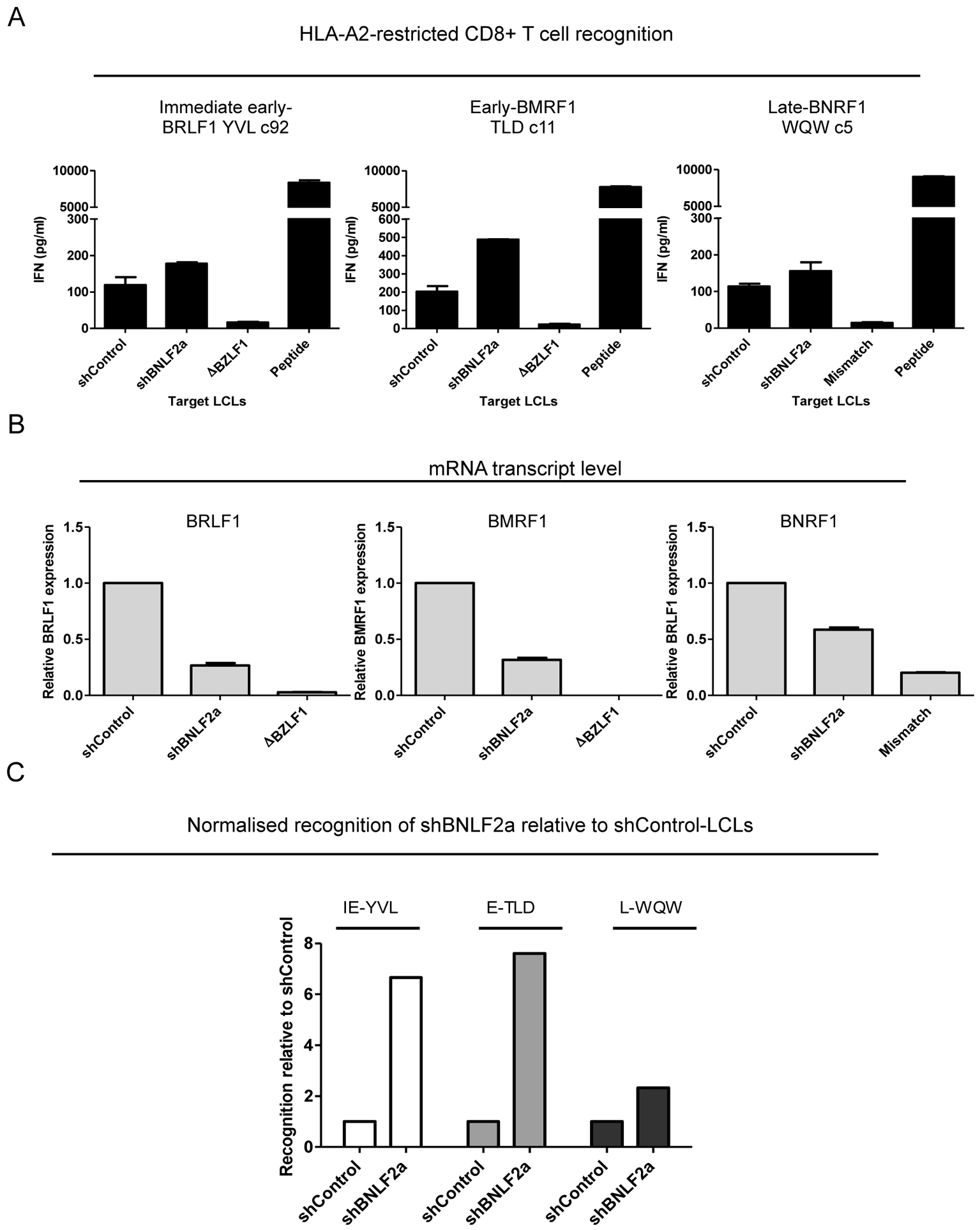

Supplement: Figure S4 — Recognition of donor 5 shBNLF2a-LCLs. (A) Recognition of donor 5 LCLs by IE-YVL, E-TLD and L-WQW specific CD8+ T cell clones. Recognition is shown as IFN-γ (pg/ml) release. Maximal experimental recognition is indicated by recognition of peptide-sensitised ΔBZLF1 LCLS. (B) Level s of IE-BRLF1, E-BMRF1 and L-BNRF1 mRNA transcripts in the target LCLs used in A. (C) Recognition of donor 5 shBNLF2a-LCLs relative to donor 5 shControl-LCLs, after normalisation of IFN-γ release against target transcript levels. (TIF) [file ppat.1004322.s004.tif]

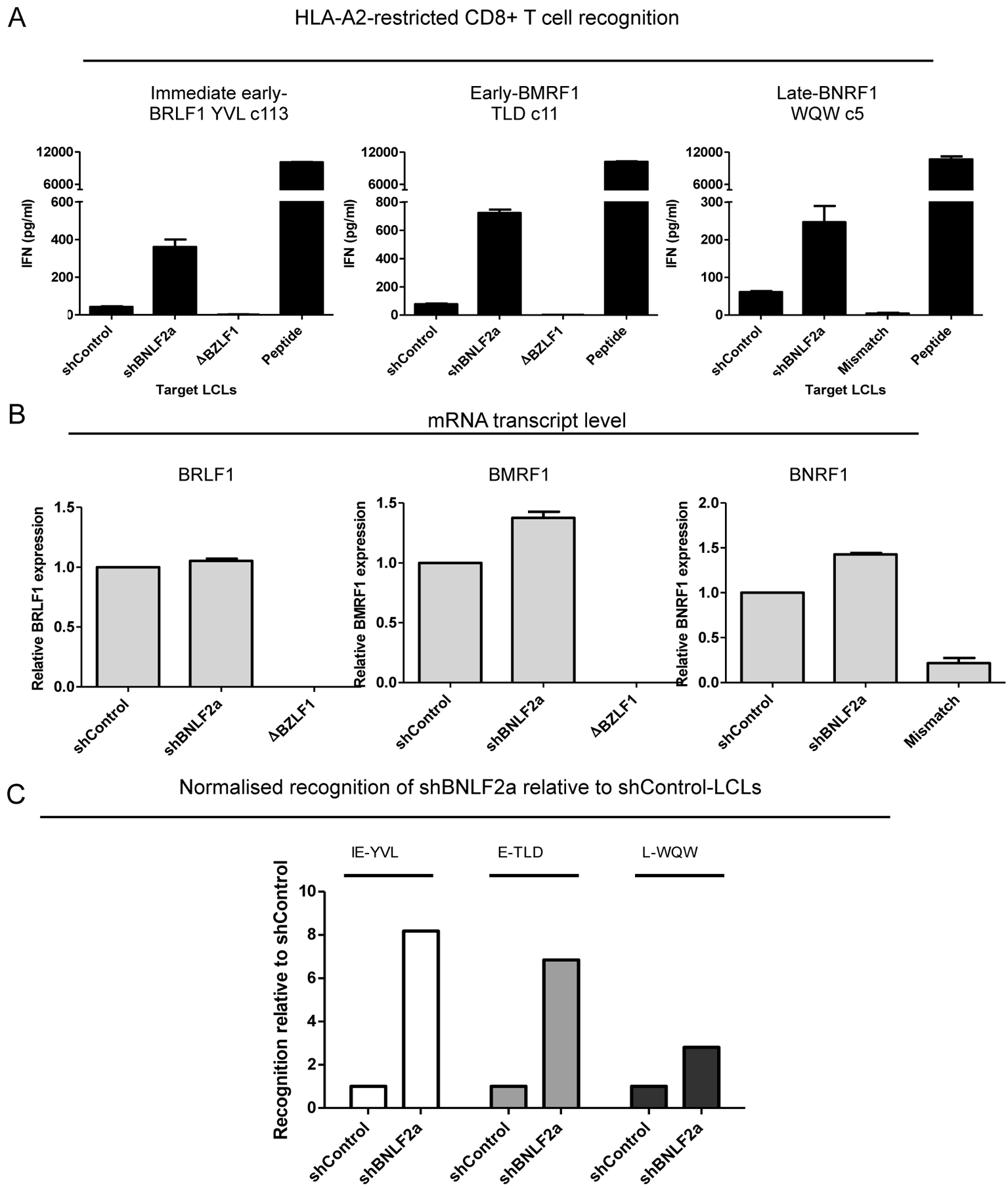

Supplement: Figure S5 — Recognition of donor 6 shBNLF2a-LCLs. A) Recognition of donor 6 LCLs by IE-YVL, E-TLD and L-WQW specific CD8+ T cell clones. Recognition is shown as IFN-γ (pg/ml) release. Maximal experimental recognition is indicated by recognition of peptide-sensitised ΔBZLF1 LCLS. (B) Levels of IE-BRLF1, E-BMRF1and L-BNRF1 mRNA transcripts in the target LCLs used in A. (C) Recognition of donor 6 shBNLF2a-LCLs relative to donor 6 shControl-LCLs, after normalisation of IFN-γ release against target transcript levels. (TIF) [file ppat.1004322.s005.tif]

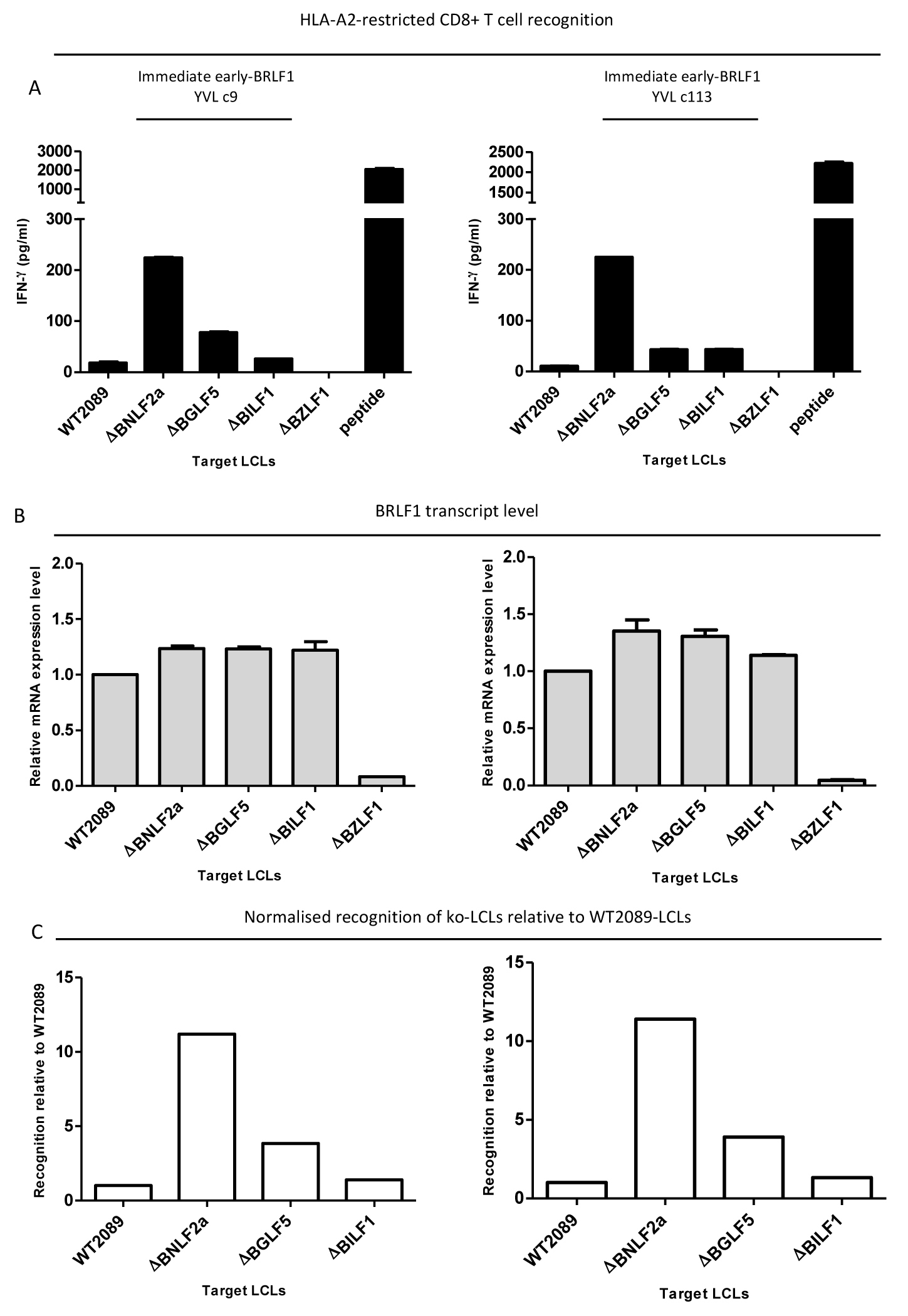

Supplement: Figure S6 — Recognition of donor 7 LCLs by IE-YVL specific CD8+ T cell clones. A) Recognition of KO-LCLs by two YVL-specific clones is shown as IFN-γ (pg/ml) release. Maximal recognition is indicated by recognition of peptide-sensitised ΔBZLF1-LCLs. B) mRNA levels of BRLF1 in target LCLs. C) Recognition of LCLs relative to WT2089-LCLs, after normalisation of IFN-γ release against transcript levels. (JPG) [file ppat.1004322.s006.jpg]

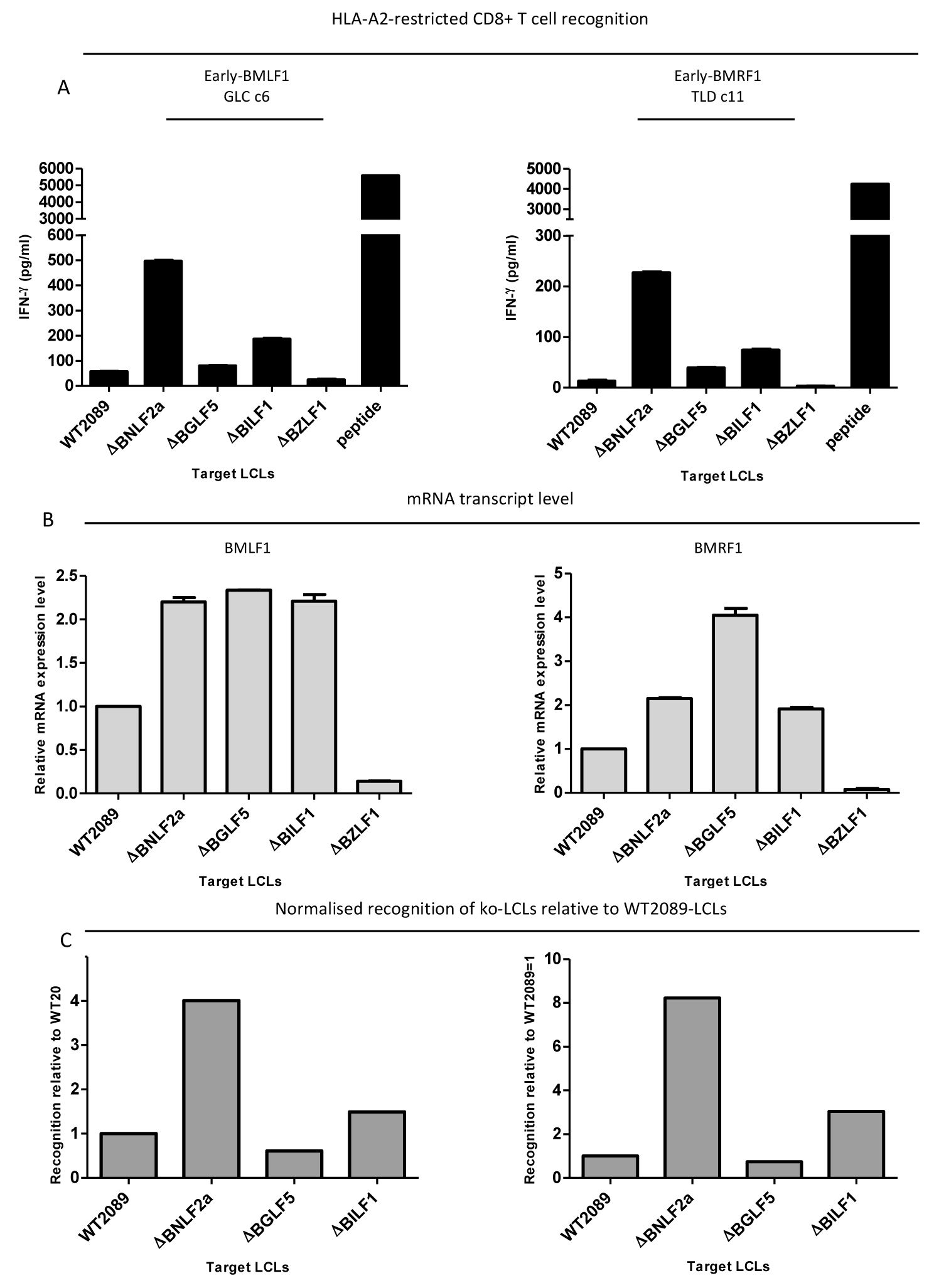

Supplement: Figure S7 — Recognition of donor 7 KO-LCLs by E-GLC and -TLD specific CD8+ T cell clones. A) Recognition of KO-LCLs shown as IFN-γ (pg/ml) release. Maximal recognition is indicated by recognition of peptide-sensitised ΔBZLF1-LCLs. B) mRNA levels of corresponding BMLF1 and BMRF1 in target LCLs. C) Recognition of LCLs relative to WT2089-LCLs, after normalisation of IFN-γ release against transcript levels. (JPG) [file ppat.1004322.s007.jpg]

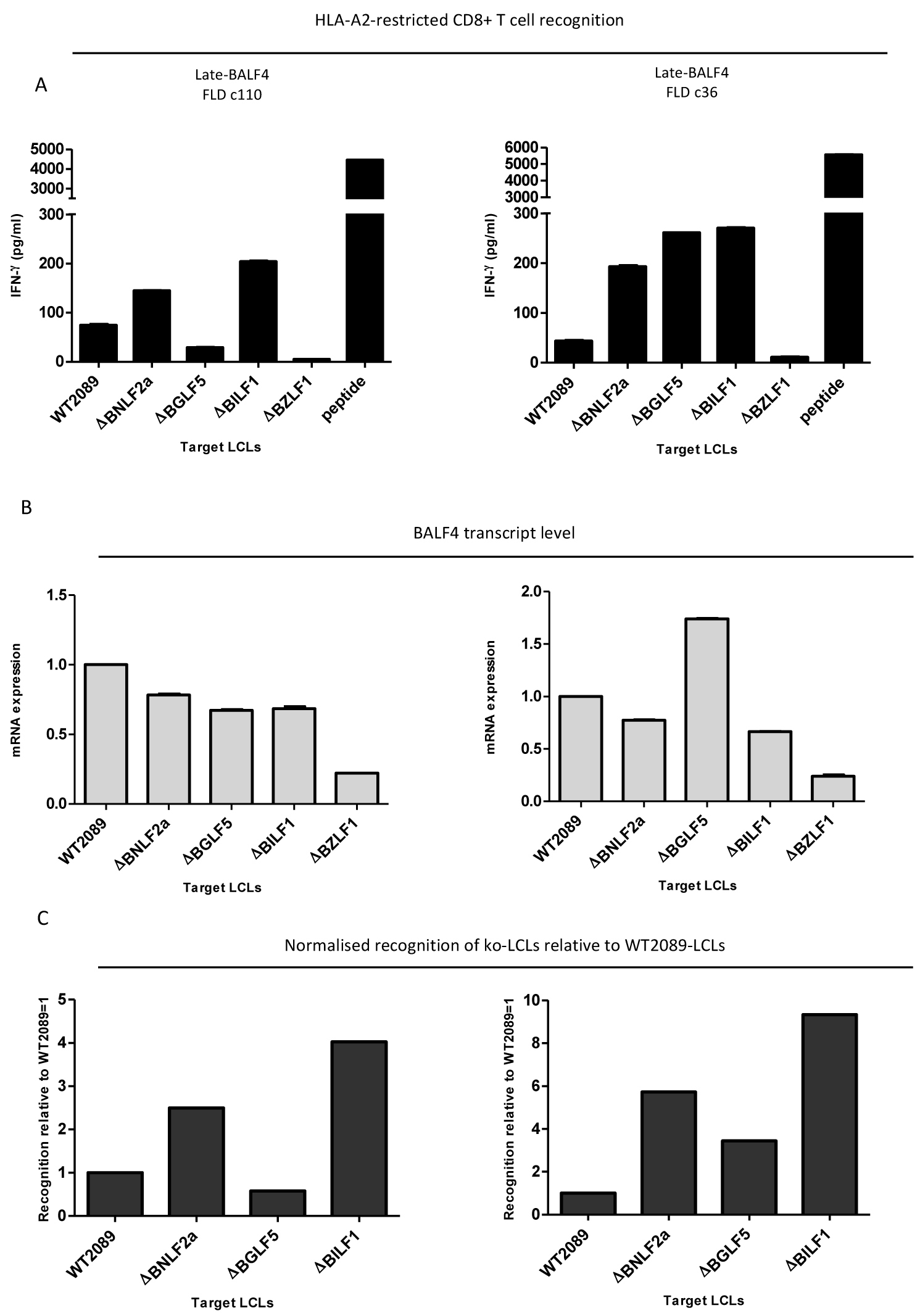

Supplement: Figure S8 — Recognition of donor 7 KO-LCLs by two L-FLD specific CD8+ T cell clones. A) Recognition of KO-LCLs shown as IFN-γ (pg/ml) release. Maximal recognition is indicated by recognition of peptide-sensitised ΔBZLF1-LCLs. B) mRNA levels of BALF4 in target LCLs. C) Recognition of LCLs relative to WT2089-LCLs, after normalisation of IFN- γ release against transcript levels. (JPG) [file ppat.1004322.s008.jpg]

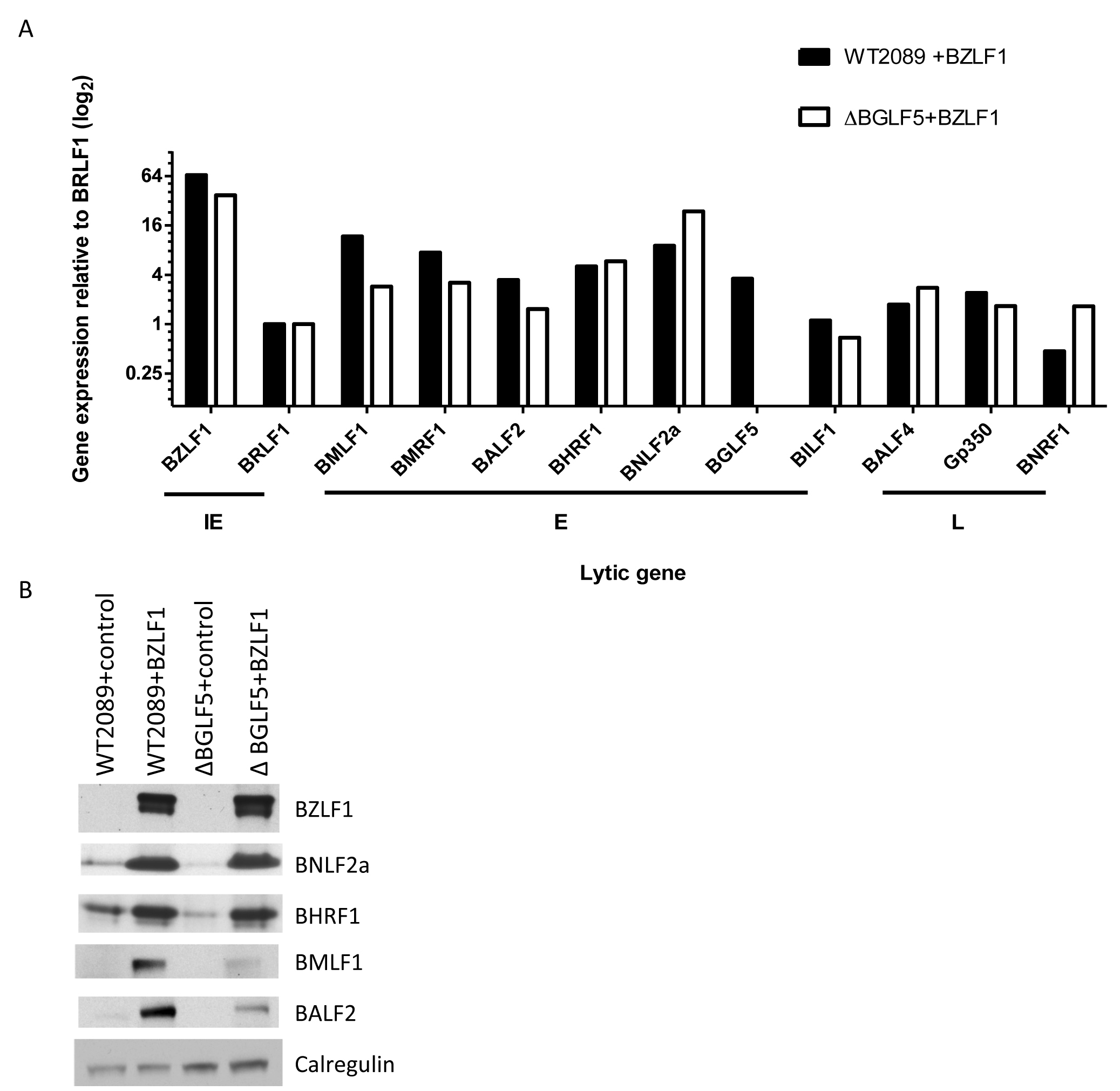

Supplement: Figure S9 — The effect of BGLF5 knockout on lytic gene and protein expression. WT2089- and counterpart ΔBGLF5 knockout-LCLs were transduced with either a pRTS-CD2-control or pRTS-CD2-BZLF1 vector. This vector carries a bidirectional doxycycline (Dox) regulatable promoter, BI-Tet, which drives the expression of BZLF1, which is able to induce lytic cycle, together with a non-functional neuronal growth factor receptor (NGFR) and green fluorescent protein (GFP) as a markers of Dox induced expression. WT2089- and ΔBGLF5-LCLs transfected with pRTS-CD2-BZLF1 or pRTS-CD2-control vector were treated for 12 hrs with Dox before selecting for induced plasmid containing cells using MACSelect LNGFR MicroBeads. (A) In one experiment, RNA was extracted from the selected cells and used to generate cDNA in order to analyse the expression of a panel of lytic cycle genes using qRT-PCR. This panel included 2 IE genes, 7 E-genes which included the immune evasion genes BNLF2a, BGLF5 and BILF1 and 3 L genes. Plotting the expression levels of each of these genes in lytically induced WT2089-LCLs (WT2089+BZLF1) alongside lytically induced ΔBGLF5-LCLs (ΔBGLF5+BZLF1) allows us to directly compare the impact of BGLF5 knockout on the expression of lytic genes. Variation in BZLF1 expression, and lytic cycle induction, between WT2089+BZLF1 and ΔBGLF5+BZLF1 LCLs were compensated by displaying of all genes relative to the expression of BRLF1 in that cell. (B) In a separate experiment, selected WT2089-control and −BZLF1 (lane 1 and 2 respectively) and ΔBGLF5-control and −BZLF1 (lane 3 and 4) transfected LCLs were also analysed by SDS-PAGE and immunoblotting with antibodies specific for the lytic cycle proteins BZLF1, BNLF2a, BHRF1, BMLF1, and BALF2, with calregulin as a loading control. (JPG) [file ppat.1004322.s009.jpg]

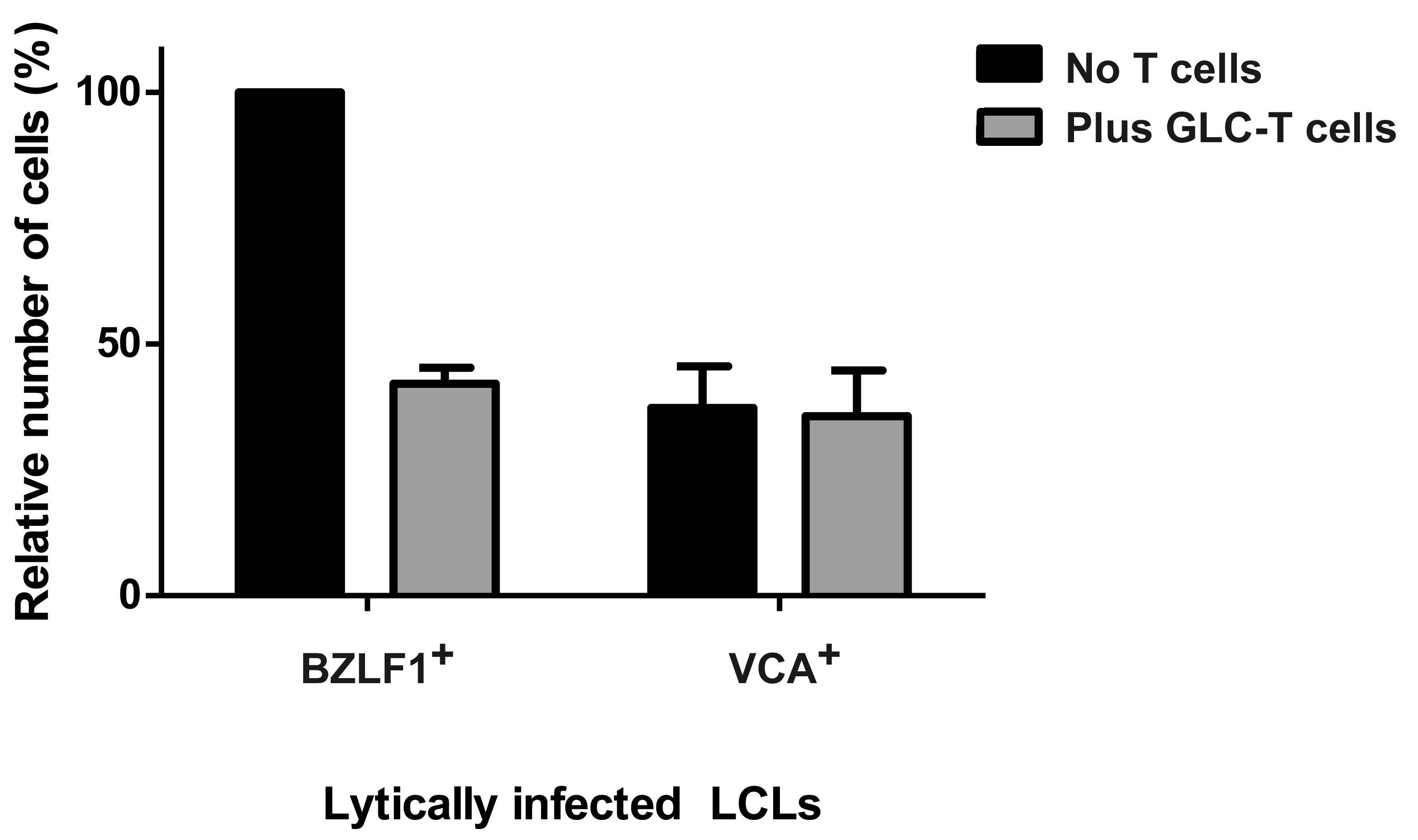

Supplement: Figure S10 — VCA+ lytically infected cells are resistant to E-antigen specific effector T cells. HLA A2 positive LCLs containing around 2% cells spontaneously in lytic cycle were co-cultured with or without GLC T cells (A2 restricted and BMLF1 specific cytotoxic CD8+ effector clone) at a ratio of 1∶1 for 16 hr. The total cell population was then harvested and stained with anti-CD19 to identify the LCL B cells, then fixed and permeabilized, and lytic LCLs were identified through intracellular for BZLF1 and VCA. The percentage of BZLF1+ B cells in the culture without GLC T cells was set as 100%, and the number of BZLF1+ or VCA+ lytic LCLs remaining following incubation with GLC-specific T cells is shown relative to this. The data show a 60% reduction in the number of BZLF1+ B cells following co-culture with GLC T cells, but no significant depletion of VCA+ B cells. (TIF) [file ppat.1004322.s010.tif]
