# Supplementary material for: Cooperation between Epstein-Barr Virus Immune Evasion Proteins Spreads Protection from CD8+ T Cell Recognition across All Three Phases of the Lytic Cycle
Source: PLoS Pathog. 2014 Aug 21;10(8):e1004322. doi: 10.1371/journal.ppat.1004322 (PMC4140850; doi:10.1371/journal.ppat.1004322)
Supplement: Table S1 — EBV lytic gene primers and probes used for qRT-PCR. (DOCX) [file ppat.1004322.s011.docx]

**Supplementary Table 1.**

**EBV lytic gene primers and probes used for qRT-PCR.**

| **EBV lytic gene assay** | **Sequence of primers** | **Sequence of probe** |
| --- | --- | --- |
| BZLF1 | F- ACGACGCACACGGAAACC  R- CTTGGCCCGGCATTTTCT | GCATTCCTCCAGCGATTCTGGCTGTT |
| BRLF1 | F- TTGGGCCATTCTCCGAAAC  R- TATAGGGCACGCGATGGAA | AGACGGGCTGAGAATGCCGGC |
| BMLF1 | F- CCCGAACTAGCAGCATTTCCT  R- GACCGCTTCGAGTTCCAGAA | AACGAGGATCCCGCAGAGAGCCA |
| BMRF1 | F- GAGGAACGAGCAGATGATTGG  R- TGCCCACTTCTGCAACGA | TGCTGTTGATGCCCAAGACGGCTT |
| BNLF2b | F- GGGTAGACCACTGGCTGGATT  R- TTGGCCTTGTTCTTTGACCTT T | CGCTACTCTCCGCCGTTCCTTCAG-A |
| BNRF1 | F- GGAGTTTCCCCCGATTCAAG  R- TCCATGCTCTCGTCCACATC | AGGGCGCAAGTTCTCCGGTACCC |
| BALF4 | F- CCAGCTTTCCTTTCCGAGTCT  R- ACACTGGATGTCCGAGGAGAA | TCCAGCCACGGCGACCTGTTC |
| BNLF2a | F- TGGAGCGTGCTTTGCTAGAG  R- GGCCTGGTCTCCGTAGAAGAG | CCTCTGCCTGCGGCCTGCC |
| BILF1 | F- TGCCTTTTGACCCAGAACATG  R- CAACGCCATACCCAAGTGAGT | TACGGAGCACATCAGGCCCAAGAACA |
| BGLF5 | F- GCAAGCCCGGGAGAGACT  R- GAGGCGACCGTTTTCGAA | CGGGTGAACATTGTGACGGCCTTC |
